# PSO-Incorporated Hybrid Artificial Hummingbird Algorithm with Elite Opposition-Based Learning and Cauchy Mutation: A Case Study of Shape Optimization for CSGC–Ball Curves

**DOI:** 10.3390/biomimetics8040377

**Published:** 2023-08-18

**Authors:** Kang Chen, Liuxin Chen, Gang Hu

**Affiliations:** 1 Unmanned System Research Institute, Northwestern Polytechnical University, Xi’an 710072, China; chenkang@nwpu.edu.cn; 2 Xi’an Jingkai No. 1 Primary School, Xi’an 710018, China; chenliuxin@163.com; 3 Department of Applied Mathematics, Xi’an University of Technology, Xi’an 710054, China; 4 School of Computer Science and Engineering, Xi’an University of Technology, Xi’an 710048, China

**Keywords:** artificial hummingbird algorithm, Cauchy mutation, elite opposition-based learning, generalized Ball curves, particle swarm optimization, shape optimization

## Abstract

With the rapid development of the geometric modeling industry and computer technology, the design and shape optimization of complex curve shapes have now become a very important research topic in CAGD. In this paper, the Hybrid Artificial Hummingbird Algorithm (HAHA) is used to optimize complex composite shape-adjustable generalized cubic Ball (CSGC–Ball, for short) curves. Firstly, the Artificial Hummingbird algorithm (AHA), as a newly proposed meta-heuristic algorithm, has the advantages of simple structure and easy implementation and can quickly find the global optimal solution. However, there are still limitations, such as low convergence accuracy and the tendency to fall into local optimization. Therefore, this paper proposes the HAHA based on the original AHA, combined with the elite opposition-based learning strategy, PSO, and Cauchy mutation, to increase the population diversity of the original algorithm, avoid falling into local optimization, and thus improve the accuracy and rate of convergence of the original AHA. Twenty-five benchmark test functions and the CEC 2022 test suite are used to evaluate the overall performance of HAHA, and the experimental results are statistically analyzed using Friedman and Wilkerson rank sum tests. The experimental results show that, compared with other advanced algorithms, HAHA has good competitiveness and practicality. Secondly, in order to better realize the modeling of complex curves in engineering, the CSGC–Ball curves with global and local shape parameters are constructed based on SGC–Ball basis functions. By changing the shape parameters, the whole or local shape of the curves can be adjusted more flexibly. Finally, in order to make the constructed curve have a more ideal shape, the CSGC–Ball curve-shape optimization model is established based on the minimum curve energy value, and the proposed HAHA is used to solve the established shape optimization model. Two representative numerical examples comprehensively verify the effectiveness and superiority of HAHA in solving CSGC–Ball curve-shape optimization problems.

## 1. Introduction

Geometric modeling mainly focuses on the representation, approximation, analysis, and synthesis of curve and surface information in computer image system environments [1]. It has been widely used in various fields such as aviation, shipbuilding, surveying and mapping, mechanical design, computer vision, bioengineering, animation, and military combat simulation [2]. The study of Ball curves and surfaces is a very important research topic in geometric modeling, mainly focusing on the geometric research of various products [3]. In 1974, Ball [4] first constructed the rational cubic parametric curves and used them as the mathematical basis for Warton’s former British Airways CONSURF fuselage surface modeling system, which was later called a Ball curve. Later, in order to extend the cubic Ball curves to higher-order generalized Ball curves, Wang–Ball curves were proposed by Wang [5] in 1987, which provided a powerful method for evaluating the higher degree curves. In 1989, Said [6] constructed Said–Ball curves, but the degree of basis functions of these curves could only be odd because the curves were derived by Hermite interpolation. In order to eliminate this limitation, Hu [7] extended the degree of the basis functions from odd to even in 1996 and defined the Said–Ball curves with arbitrary times. Subsequently, Othman [8], Xi [9], and Ding [10] independently discussed the dual basis functions and their applications to generalized Said–Ball curves. In 2004, Jiang [11] studied the dual basis functions of Wang–Ball curves and their applications, which further improved the theory of Wang–Ball curves.

Compared with the Bézier curves, generalized Ball curves have the same basic properties as the Bézier curves, but at the same time, they also have faster calculation speeds and better calculation efficiency [12]. Therefore, generalized Ball curves have increasingly become an important research hotspot in the field of curve shape design. However, the control points and the corresponding basis functions of the curves are the only basis for determining the shape of generalized Ball curves. If changes are to be made to the shape of the curves, the position of the control points must be adjusted, which makes the adjustment of the curve shape extremely inflexible. In order to improve the flexibility of curve shape adjustment, many scholars have constructed generalized Ball curves with a single shape parameter that can control the shape of the curves by adjusting the value of the shape parameter.

In 2000, Wu [13] proposed two new types of generalized Ball curves, namely *n*-th Said–Bézier–Ball (SBGB) curves and Wang–Said–Ball (WSGB) curves. The SBGB curves are between Said–Ball curves and Bézier curves, and the WSGB curves are between Wang–Ball curves and Said–Ball curves. Both of these new curves adjust the shape of the curves by introducing position parameters. On the basis of the cubic Ball curves, Wang [14] further proposed the cubic Ball curves with shape control parameter λ in 2008. In 2009, Wang [15] increased the degree of cubic Ball curves, proposed quartic Wang–Ball curves with one shape control parameter λ, and the shape of the curves can be modified by controlling the value of the λ. In the same year, Hu [16] constructed the 2*m* + 2 degree generalized Said–Ball curves with a single shape parameter λ, and the ideal shape could be obtained by changing the value of the shape parameter λ. In 2011, Yan [17] constructed two new types of quintic generalized Ball curves with a shape parameter. In 2013, Xiong et al. [18] proposed *n*-th degree Wang–Ball curves with a shape parameter λ, which can control the shape of the curves by changing the parameter λ, especially when λ=2, these curves can be degenerated into traditional *n*-th degree Wang–Ball curves.

The above generalized Ball curves with a single parameter solve the limitation that the shape of traditional Ball curves cannot be adjusted to some extent. However, because they only take a single shape parameter, the curves can only simply swing, and the adjustment of the curve shape is still inflexible, making it difficult to meet people’s daily expectations and requirements for curve flexibility.

In order to make the generalized Ball curve shape adjustment with higher flexibility, in 2011, Liu [19] constructed the quadratic Q-Ball curves with two parameters, λ and μ, which improved the flexibility of curve shape adjustment. Subsequently, in 2012, Huang et al. [20] proposed the quartic Wang–Ball curves, which have two shape parameters, α and β, to better control the shape of the curves through the coordination of α and β. Compared with the generalized Ball curves with a single shape parameter, the flexibility of the generalized Ball curves with two shape parameters has significantly improved, but there are still some limitations in adjusting the local shape of the curves. Therefore, Hu [21] constructed new generalized cubic Ball basis functions in 2021. On this basis, SGC–Ball curves with adjustable shape parameters were proposed, which adjusted the shape of the curves through the joint adjustment of multiple parameters. Meanwhile, in 2022, Hu et al. [22] proposed CG–Ball curves with multiple shape control parameters, which can flexibly control the shape of the curves through the coordination of multiple shape parameters. In the construction of curve modeling, the SGC–Ball curves can achieve satisfactory results, but the curve graph in real life is usually very complicated, and it is difficult to build the ideal curves with the single SGC–Ball curve. Therefore, in order to meet the design requirements of more complex geometric product modeling, this paper studies the smooth splicing continuity conditions of two adjacent SGC–Ball curves, G^1^ and G^2^. Secondly, the complex combined SGC–Ball curves (CSGC–Ball curves for short) are constructed on the basis of the SGC–Ball curves. It is worth noting that the curves have a global shape parameter and some local shape parameters. By adjusting the global or local shape parameters, the overall or local shape of the curves can be adjusted more flexibly. The CSGC–Ball curves can be used to construct complex curves more flexibly in engineering.

In addition, the shape optimization problem of curves has also become a very important research hotspot in CAGD. In order to construct the CSGC–Ball curves with ideal shapes, the CSGC–Ball curve-shape optimization problem with the overall G^1^ and G^2^ continuity conditions is also studied in this paper. The energy method is a classical method for establishing curve-shape optimization models. In 1987, Terzopoulos et al. [23] first combined computer graphics with physics-based energy models, and then the energy method was widely used to study different types of curve and surface optimization problems, as can be seen in references [24,25,26,27]. Therefore, in this paper, the minimum curve bending energy value is taken as the objective function to establish CSGC–Ball curve-shape optimization models with G^1^ and G^2^ continuity, respectively. By solving the shape optimization models and obtaining the optimal parameter values, the most ideal curve shape can be obtained. However, due to the fact that the objective function of the established shape optimization models is highly nonlinear, has multiple shape parameters, and has high computational complexity, solving the CSGC–Ball curve optimization models using traditional optimization methods is very complex and challenging. Solving the shape optimization model also solves the optimization problem. The Meta-Heuristic Algorithm (MA) has become a popular method for solving complex optimization problems in different fields due to its simplicity and flexibility [28]. Therefore, this paper considers using MA to solve the CSGC–Ball curve-shape optimization models.

MA is an optimization method established by simulating different natural phenomena in nature. It has the advantages of flexibility, simplicity, and wide application [29]. According to No-Free-Lunch (NFL) [30], no single MA can solve all optimization problems, so there are many meta-heuristics. According to the different sources of design inspiration, MA is mainly divided into the following four categories: Evolutionary Algorithm (EA), Physics-based Algorithm (PA), Human-based Algorithm (HA), and Swarm Intelligence (SI) [31], as shown in Table 1.

EA is a kind of algorithm used to simulate the evolutionary behavior of organisms in nature. The most classic EA is the Genetic Algorithm (GA) [32], inspired by Darwin’s evolutionary theory, which seeks the optimal solution for the population according to the law of survival of the fittest. In addition, popular EA also includes Differential Evolution (DE) [33], Genetic Programming (GP) [34], the Species Co-evolutionary Algorithm (SCEA) [35], etc. PA is designed according to physical laws or chemical reaction principles, and the Simulated Annealing (SA) [36] algorithm is the most typical and widely used example. Others include the Gravity Search Algorithm (GSA) [37] based on Newton’s law of universal gravitation, the Sine Cosine Algorithm (SCA) [38] that simulates the sine cosine function to find the optimal solution, the Archimedes Optimization Algorithm (AOA) [39] designed based on Archimedes’ principle, the Crystal Structure Algorithm (CryStAl) [40], and Smell Agent Optimization (SAO) [41] that simulates the interactions between a smell agent and an object evaporating a smell molecule. HA is related to human social behavior. The most classic algorithm is Teaching–Learning-Based Optimization (TLBO) [42], based on the improvement of class levels. Other algorithms include Social Group Optimization (SGO) [43], Student Psychology Based Optimization (SPBO) [44], Bus Transportation Algorithm (BTA) [45], Alpine Skiing Optimization (ASO) [46], etc.

SI is the most popular branch of MA used to simulate the collective behavior of social animals in nature. Particle Swarm Optimization (PSO) [47] is the most classic SI, inspired by the social behavior of birds and often used to solve various global optimization problems. Famous SI algorithms also include, but are not limited to, Ant Colony Optimization (ACO) [48], based on the collective behavior of ant colony, Moth–Flame Optimization (MFO) [49,50,51], the Grey Wolf Optimizer (GWO) [52] simulating the cooperative hunting behavior of gray wolves, the Whale Optimization Algorithm (WOA) [53], Harris Hawk Optimization (HHO) [54], the Black Widow Algorithm [55,56], the Seagull Optimization algorithm (SOA) [57], the Salp Swarm Algorithm (SSA) [58,59], the African Vultures Optimization Algorithm (AVOA) [60], the Dwarf Mongoose Optimization Algorithm (DMOA) [61], the Pelican Optimization Algorithm (POA) [62], Golden Jackal Optimization (GJO) [63], the Artificial Hummingbird Algorithm (AHA) [64], etc. Among them, AHA is a recently proposed bionic MA that is inspired by the intelligent foraging behaviors, special flight skills, and amazing memory function of hummingbirds. Hummingbirds have three unique flight skills: axial, diagonal, and omnidirectional flight. These skills are flexibly and alternately used in its three foraging behaviors. The migration foraging strategy provides the algorithm with powerful exploration capabilities, territorial foraging improves population diversity and avoids the possibility of the algorithm falling into local optima, and guided foraging creates an intelligent balance between exploration and exploitation. In addition, the visit table was established to simulate the powerful memory abilities of hummingbirds.

The performance of AHA is competitive with other well-known algorithms [64]. In 2022, Ramadan [65] made improvements on the basis of the original AHA and proposed an adaptive opposition artificial hummingbird algorithm, referred to as AOAHA, which improved the performance of the AHA and applied it to solve an accurate photovoltaic model of the solar cell system. In the same year, Mohamed [66] proposed the Artificial Hummingbird Optimization Technology (AHOT) to solve the parameter identification problem of lithium-ion batteries for electric vehicles. Meanwhile, in [67], Sadoun et al. used a machine learning method based on AHA to predict the effect of the tribological behavior of in situ-synthesized Cu-Al_2_O_3_ nanocomposites. In 2022, AHA was used in [68] to solve the planning optimization problem of multiple renewable energy integrated distribution systems with uncertainty, and the optimization results were better.

Compared with other advanced meta-heuristic algorithms, AHA can quickly and accurately find the global optimal solution and has certain applicability and competitiveness in terms of computational accuracy and time. However, due to the standard AHA being designed as simply as possible, there are still certain limitations when solving complex optimization problems, such as slow iteration speed, low diversity, and the tendency to converge prematurely. In order to make the original AHA more competitive, another goal of this paper is to propose a hybrid artificial hummingbird algorithm (HAHA) based on the standard AHA, that is, the elite opposition-based learning strategy [69], the PSO strategy [47], and the Cauchy mutation strategy [70] combined with the original AHA. Three strategies work together to increase the optimization ability and overall performance of AHA. The proposed HAHA algorithm is tested on 25 benchmark functions and the CEC 2022 test suite, and it is verified that the proposed HAHA shows good competitiveness in solving global optimization problems. Therefore, the proposed HAHA is used to solve the established CSGC–Ball curve-shape optimization models. The main contributions of this paper are as follows:

(1) The smooth splicing continuity conditions of adjacent SGC–Ball curves G^1^ and G^2^ are derived, and the combined SGC–Ball curves with global and local shape parameters are constructed, called CSGC–Ball curves, which verify that the CSGC–Ball curves have better shape adjustability.
(2) Based on the original AHA, an enhanced AHA (HAHA) is proposed by combining three strategies to effectively solve complex optimization problems. To demonstrate the superiority of HAHA, numerical experiments are compared with other advanced algorithms on the 25 benchmark functions and the CEC 2022 test set. The superiority and practicality of the proposed HAHA have been comprehensively verified.
(3) According to the minimum energy, the CSGC–Ball curve optimization model is established. The proposed HAHA is used to solve the established model, and the results are compared with those of other algorithms. The results demonstrate that the proposed HAHA is effective in solving the CSGC–Ball curve-shape optimization model.

**Table 1:** Classification of some advanced MAs.

Type	Algorithm	Year	Reference
Evolutionary Algorithm (EA)	Genetic Algorithm (GA)	1992	[32]
	Differential Evolution (DE)	1997	[33]
	Genetic Programming (GP)	1992	[34]
	Simulated Annealing (SA)	1983	[36]
Physics-based Algorithm (PA)	Gravity Search Algorithm (GSA)	2009	[37]
	Sine Cosine Algorithm (SCA)	2016	[38]
	Archimedes Optimization Algorithm (AOA)	2020	[39]
	Crystal Structure Algorithm (CryStAl)	2021	[40]
	Smell Agent Optimization (SAO)	2021	[41]
Human-based Algorithm (HA)	Teaching-Learning-Based Optimization (TLBO)	2012	[42]
	Psychology Based Optimization (SPBO)	2020	[44]
	Bus Transportation Algorithm (BTA)	2019	[45]
	Alpine Skiing Optimization (ASO)	2020	[46]
Swarm Intelligence (SI)	Ant Colony Optimization (ACO)	1995	[48]
	Grey Wolf Optimizer (GWO)	2014	[52]
	Whale Optimization Algorithm (WOA)	2016	[53]
	Harris Hawk Optimization (HHO)	2019	[54]
	African Vultures Optimization Algorithm (DMOA)	2021	[60]
	Dwarf Mongoose Optimization Algorithm (DMOA)	2022	[61]
	Pelican Optimization Algorithm (POA)	2022	[62]
	Golden Jackal Optimization (GJO)	2022	[63]
	Artificial Hummingbird Algorithm (AHA)	2022	[64]

The rest of the paper is structured as follows: 

Section 2 introduces the proposed HAHA in detail. Numerical experiments to evaluate the performance of the proposed HAHA are given in Section 3. Section 4 introduces the constructed combined SGC–Ball curves and studies the G^1^ and G^2^ continuous splicing conditions for the SGC–Ball curves. In Section 5, the CSGC–Ball curve-shape optimization models are established based on minimum energy, and the detailed process of solving the shape optimization model using the proposed HAHA is given. Section 6 summarizes the paper and provides future research directions.

## 2. Hybrid Artificial Hummingbird Algorithm

### 2.1. Basic Artificial Hummingbird Algorithm

**Figure 1:**
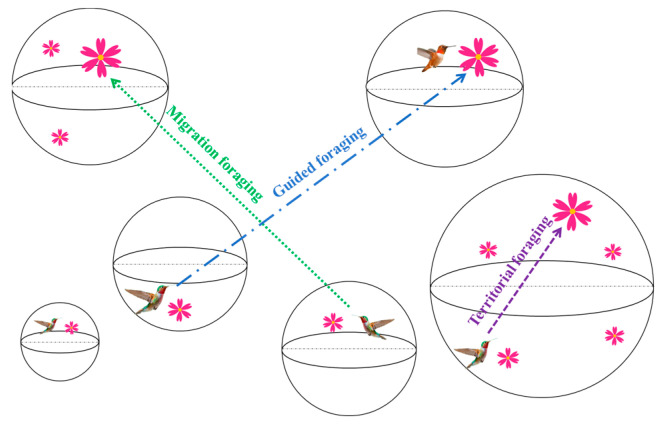
Three foraging behaviors of hummingbirds.

The Artificial Hummingbird Algorithm (AHA) [64] is a novel bionic MA proposed in 2021, inspired by the unique flight skills, intelligent foraging strategies, and strong memory capacity of hummingbirds. Hummingbirds are the smallest but most intelligent birds in the world. They have three special flight skills and three intelligently adjusted foraging strategies. Three foraging behaviors of hummingbirds are shown in Figure 1. Meanwhile, most notably, they have a strong memory, so AHA constructed the visit table to simulate the unique memory ability of hummingbirds for food sources.

#### 2.1.1. Initialization

AHA uses the random initialization method to generate hummingbird population ***X***, and randomly places *n* hummingbirds on *n* food sources, as described by Equation (1):

(1) $$x_{i}=Lb+r\cdot (Ub-Lb),\;\;\;i=1,\ldots ,n,$$

where ***X*** = {*x*_1_,…,*x_n_*} is the hummingbird population, *n* represents the population size, *x_i_* is the location of the *i*-th food source, *r* is a *d*-dimensional random vector in [0,1], and *Ub =* {*ub*_1_,…,*ub_d_*} and *Lb =* {*lb*_1_,…,*lb_d_*} are upper bounds and lower bounds, respectively. The visit table is initialized by Equation (2):

(2) $$VT_{i,j}=\left\{\begin{array}{l} null\;\;\;\;if\;i=j\\ 0\;\;\;\;\;\;\;if\;i\neq j \end{array}\right.\;i=1,\ldots ,n;\;j=1,\ldots ,n,$$

where *VT_i,j_* is the visit level, indicating the time period when the *i*-th hummingbird did not reach the *j*-th food source; *null* indicates the hummingbird visited the food source.

#### 2.1.2. Guided Foraging

**Figure 2:**
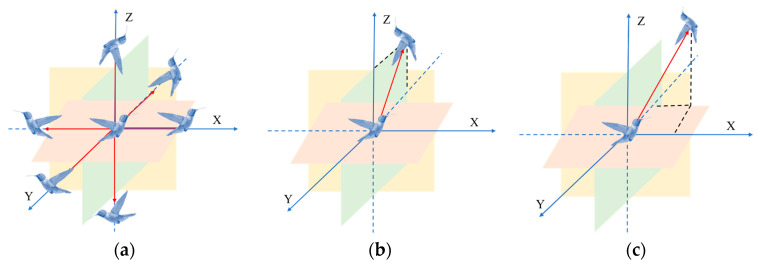
Three special flight abilities of hummingbirds: (**a**) axial fight behavior; (**b**) diagonal flight behavior; (**c**) omnidirectional flight behavior.

In the process of foraging, hummingbirds have three special flight skills: axial, diagonal, and omnidirectional flight. Use the direction switching vector ***D*** to determine which flight skill the hummingbird chooses. Figure 2 describes the three flight behaviors in three-dimensional space. Figure 2a shows axial flight, in which the hummingbird can choose to fly in an arbitrary direction of the coordinate axis; Figure 2b reflects diagonal flight, in which the hummingbird can fly from any angle of the coordinate axis to its diagonal position; and Figure 2c demonstrates omnidirectional flight, in which the hummingbird can fly in any direction.

In *d*-dimensional space, the expressions for simulating the axial, diagonal, and omnidirectional flight of hummingbirds are expressed by Equations (3)–(5), respectively.

(3) $$D^{\left(i\right)}=\left\{\begin{array}{l} 1,\;if\;i=randi([1,d])\\ 0,\;else \end{array}\right.\;\;i=1,\ldots ,d,$$

(4) $$D^{\left(i\right)}=\left\{\begin{array}{l} 1,\;if\;i=P(j),j=[1,q],P=randprem(q)\\ 0,\;else \end{array}\right.,$$

(5) $$D^{\left(i\right)}=1\;\;i=1,\ldots ,d,$$

where *randi*([1,*d*]) is a randomly generated integer in [1,*d*], $q\in [1,\left\lceil rand\cdot (d-2)\right\rceil +1]$, and *randprem*(*q*) represents generating a random arrangement of integers from 1 to *q*.

Hummingbirds will rely on the alternation of three flight skills to reach the target food source and use Equation (6) to simulate guided foraging to obtain the position of candidate food source *v_i_*.

(6) $$v_{i}(t+1)=x_{i,aim}(t)+A\cdot D\cdot (x_{i}(t)-x_{i,aim}(t)),$$

where *v_i_*(*t* + 1) is the position of the candidate solution in iteration *t* + 1, and *x_i_*(*t*) is the *i*-th food source in iteration *t*. In addition, *x_i_*_,*aim*_(*t*) is the location of the target food source where the *i*-th hummingbird will be located. *A*~*N*(0,1) is the guiding parameter that obeys the normal distribution.

The position of the *i*-th food source of the hummingbird is updated by Equation (7).

(7) $$x_{i}(t+1)=\left\{\begin{array}{l} x_{i}(t),\;if\;f(x_{i}(t))\leq f(v_{i}(t+1))\\ v_{i}(t+1),\;f(x_{i}(t))>f(v_{i}(t+1)) \end{array}\right.,$$

where *f*(*x_i_*(*t*)) and *f*(*v_i_*(*t* + 1)) represent the nectar replenishment rates of hummingbird food sources and candidate food sources, respectively; that is, the fitness value of the function.

The visit table simulates the unique memory ability of hummingbirds and is used to store important time information for accessing food sources. Each hummingbird can find the food source they are going to visit based on the information on the visit table. They prefer the food source with the highest visit level, but if multiple food sources have the same visit level, the food source with the highest supplement rate will be selected. In each iteration, after the hummingbird selects the target food source through guided foraging by Equation (6), the visit table will be updated accordingly; for the update details of the visit table, refer to reference [64]. 

#### 2.1.3. Territorial Foraging

When hummingbirds visit target candidate solutions, they will move into adjacent territories in search of new food sources that may be better candidates than existing ones. The mathematical expression for simulating the territorial foraging strategy of hummingbirds is:

(8) $$v_{i}(t+1)=x_{i}(t)+B\cdot D\cdot x_{i}(t),$$

where *v_i_*(*t* + 1) is the position of the candidate food source obtained by hummingbird *i* through territorial foraging in *t* + 1 iterations, and *B*~*N*(0,1) represents the territorial parameter obeying the normal distribution. Hummingbirds update the visit table after performing territorial foraging.

#### 2.1.4. Migration Foraging

Hummingbirds tend to migrate further afield to feed when there is a shortage of food in the areas they visit. The migration coefficient, *M*, is the value given to determine whether the hummingbird is migrating. If the number of iterations exceeds *M*, the hummingbird with the worst fitness value will randomly migrate to any randomly generated food source in the search space for foraging. The migration foraging behavior of hummingbirds from the food source with the worst nectar replenishment rate to the randomly generated food source can be expressed as

(9) $$x_{wor}(t+1)=Low+r\cdot (Up-Low),$$

where *x_wor_*(*t* + 1) is the food source with the worst nectar supplementation in the hummingbird population, and *r* is the random vector in [0,1]. Hummingbirds will update the visit table after migration and foraging. Here, the migration coefficient *M* = 2*n*. The pseudo-code of the original AHA can be found in reference [64].

### 2.2. Hybrid Artificial Hummingbird Algorithm

Compared with other commonly used MAs, AHA can quickly find the global optimal solution and has certain applicability and potential for solving global optimization problems. However, the original AHA still has some limitations in solving some complex optimization problems, such as low algorithm accuracy and the tendency to fall into local optima. In order to make the original AHA more competitive, a new hybrid artificial hummingbird algorithm (HAHA) is proposed in this study, which makes the following three improvements based on the original AHA. Firstly, the introduction of a light opposition-based learning strategy in the guided foraging process helps to improve hummingbirds’ search ability, which can effectively improve the exploration ability of standard AHA. Secondly, the introduction of the PSO strategy in the exploitation stage of AHA helps hummingbirds learn from individuals with good fitness values in the population, accelerates the convergence speed, and improves the accuracy of the algorithm. Lastly, the Cauchy mutation strategy is introduced into the migration foraging of hummingbirds to expand the range of mutation, which helps the algorithm get out of stagnation and improve the search efficiency of the original AHA.

#### 2.2.1. Elite Opposition-Based Learning

AHA communicates information within the population by the visit table, which largely limits the search range of hummingbirds and easily makes the population fall into the local optimum, thereby affecting the accuracy of the solution. In order to improve the possibility of individuals approaching the optimal value in the exploration stage, the elite opposition-based learning (EOL) strategy [69] is introduced on the basis of the original AHA to improve the exploration ability of the algorithm. EOL is an innovative search method in intelligent computing. The main idea is as follows: first, the hummingbird individual with the best fitness value is regarded as the elite individual $e(t)=\{e^{1}(t),e^{2}(t),\ldots e^{d}(t)\}$; the elite individual is used to generate the opposition solution to the current solution; and the better solution is selected instead of the original solution. Then, the elite opposition-based solution $x_{i,elite}(t)=\{x_{i,elite}^{1}(t),x_{i,elite}^{2}(t),\ldots x_{i,elite}^{d}(t)\}$ of the hummingbird individual $x_{i}(t)=\{x_{i}^{1}(t),x_{i}^{2}(t),\ldots x_{i}^{d}(t)\}$ can be defined by Equation (10):

(10) $$x_{i,elite}^{j}(t+1)=rand(0,1)\cdot (ea(t)+eb(t))-x_{i}^{j}(t),\;i=1,\ldots ,n,\;j=1,\ldots ,d$$

where $ea(t)=\min(e^{j}(t))$,$eb(t)=\max(e^{j}(t))$, *j* = 1,…,*d*.

In guiding the foraging stage, the EOL strategy can better enable hummingbirds to forage for food sources with the highest nectar replenishment rates, improve their exploration abilities, enhance population diversity, and reduce the probability of falling into the local optimum, thereby improving the global search ability of hummingbird populations.

#### 2.2.2. PSO Strategy

In the exploitation stage, hummingbirds need to search for novel food sources and then select the food source with the highest nectar supplement rate as the object to be visited, according to the visit table. However, it does not consider learning from hummingbirds with good fitness values in the population, which still has certain limitations. PSO [47] is an optimization algorithm proposed by Eberhart and Kennedy in 1995 that has the advantages of fast convergence speed and easy implementation. The speed update equation is shown in Equation (11):

(11) $$\begin{array}{l} v_{i,p}(t+1)=w\cdot x_{i}(t)+c_{1}\cdot rand\cdot (x_{i,pbest}(t)-x_{i}(t))+\\ \;\;\;\;c_{2}\cdot rand\cdot (x_{gbest}-x_{i}(t))+b\cdot D\cdot x_{i}(t) \end{array},$$

where *c*_1_ and *c*_2_ are learning factors with a value of 2, *x_i_*_,*pbest*_ is the local optimal solution, and *x_gbest_* represents the global optimal solution.

(12) $$w=(w_{ini}-w_{end})\frac{\left(T-t\right)}{T}+w_{end},$$

in Equation (12), *w* is the inertia factor, and *w_ini_* = 0.4 and *w_end_* = 0.9 are the initial and final inertia weights, respectively. With the increase in iterations, *w* shows a decreasing trend.

The speed update formula in PSO is introduced into the exploitation stage of standard AHA so that hummingbirds learn from individuals with good fitness values in the population, which increases the convergence speed and accuracy of the solution of the original AHA.

#### 2.2.3. Cauchy Mutation Strategy

In AHA, the main purpose of hummingbirds choosing migration foraging is to enhance the exploration skills of the algorithm. When the number of iterations exceeds the migration coefficient *M*, the hummingbirds with the worst nectar replenishment rate will migrate to randomly generated food sources, which realizes the global exploration of the algorithm. However, in the experiments, it was found that it is still easy for the standard AHA to fall into local optimization.

In this paper, the Cauchy mutation strategy [70] is introduced to generate larger disturbances near randomly generated hummingbird individuals to improve the mutation ability of the hummingbird population, so as to improve the global search ability of the algorithm and increase the mutation range, thereby preventing the algorithm from falling into a local optimal state prematurely. The Cauchy distribution is the unique continuous probability distribution; the one-dimensional Cauchy distribution density function is shown in Equation (13):

(13) $$f(x;\delta ,\mu )=\frac{1}{\pi }\frac{\delta }{\delta^{2}+{\left(x-\mu \right)}^{2}},\;x\in (-\infty ,\infty ),$$

when *δ* = 0 and *μ* = 1, the Cauchy density function is defined by Equation (14):

(14) $$f(x;0,1)=\frac{1}{\pi (x^{2}+1)},\;x\in (-\infty ,\infty ),$$

the standard Cauchy distribution formula is described by Equation (15):

(15) $$cauchy(0,1)=\tan(\pi (\xi -\frac{1}{2})),\;\xi \in U[0,1].$$

Equation (16) is used to perform Cauchy mutation processing on randomly generated food sources in migration foraging:

(16) $$x_{cauchy}(t+1)=x_{wor}(t+1)+r\times cauchy(0,1),$$

where *cauchy*(0,1) is the Cauchy mutation operator.

The Cauchy mutation strategy is introduced in the exploration stage of the original AHA to ensure that hummingbird individuals learn from other random individuals in the population, which expands the search range of the hummingbird population, increases the diversity of the population, thereby effectively improving the AHA’s accuracy and convergence speed.

#### 2.2.4. Detailed Steps for the Proposed HAHA

**Figure 3:**
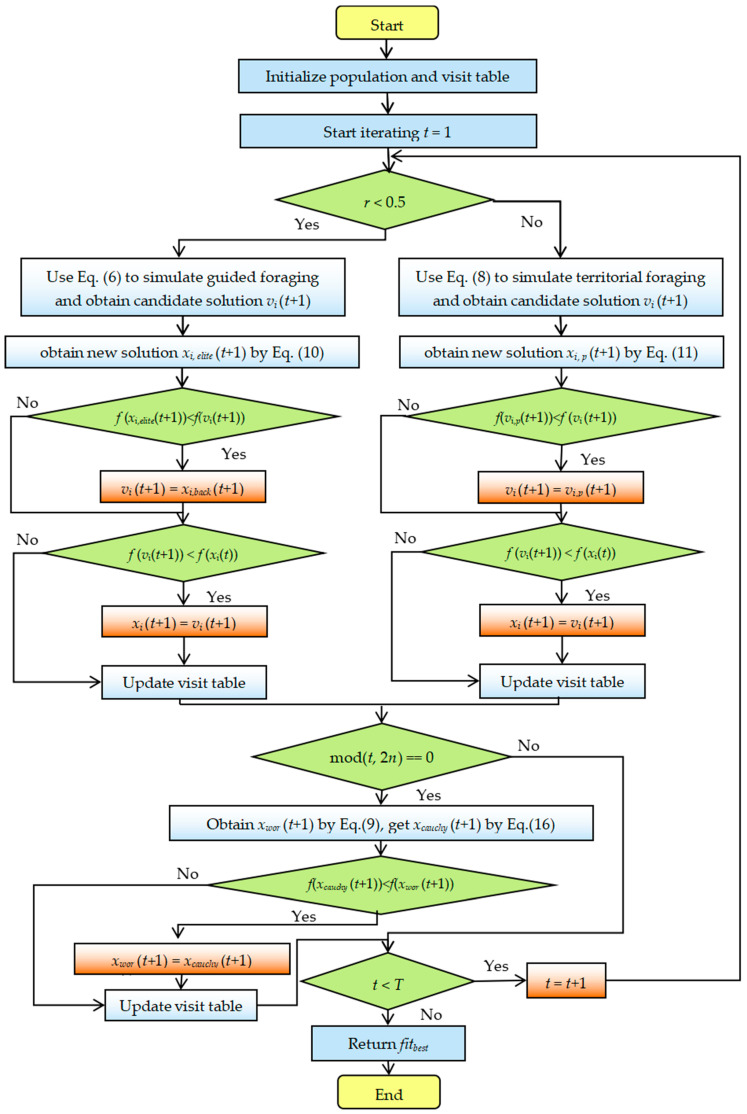
Flowchart of the proposed HAHA.

This part details the specific steps of the proposed HAHA. In order to improve the performance of the original AHA, combined with the EOL, the PSO strategy, and the Cauchy mutation, HAHA is proposed. In order to describe the process of the proposed HAHA in more detail, Figure 3 summarizes the specific implementation steps and flow chart of the proposed HAHA.

### 2.3. Computational Complexity of the Proposed HAHA

Computational complexity is one of the significant indicators used to evaluate the efficiency of an algorithm, including space complexity and time complexity. The computational complexity of the proposed HAHA is related to algorithm initialization (*Init*), the individual fitness value (*FV*) in each iteration, *D*, *n,* and *T*. In this paper, “*Oh*” represents the computation complexity of the algorithm. Initialization is to assign values to each dimension of the hummingbirds, so the computation complexity is expressed as *Oh*(*nD*). HAHA needs to calculate the individual fitness value in each iteration, so the computational complexity can be defined as *Oh*(*T‧FV‧n*). HAHA introduces EOL in guided foraging (*gui fora*), which increases the computational complexity of AHA. The computational complexity of this stage is *Oh*(*TnD*/2 + *TnD*/2). The PSO strategy is introduced in territorial foraging (*ter fora*), and the computational complexity of this stage is defined as *Oh*(*TnD*/2 + *TnD*/2). The Cauchy mutation strategy is introduced in migration foraging (*mig fora*), so the computational complexity is *Oh*((*TnD* + *TnD*)/(2*n*)). Therefore, the overall computational complexity of the proposed HAHA can be expressed by Equation (17):

(17) $$\begin{array}{l} Oh(HAHA)=Oh(problem\;\;definition)+Oh(Init)+Oh(t(FV))+Oh(t(gui\;fora))\\ \;\;\;\;\;\;+Oh(t(ter\;fora))+Oh(t(mig\;fora))\\ \;\;\;\;=Oh(1+nD+T\cdot FV\cdot n+\frac{TnD+TnD}{2}+\frac{TnD+TnD}{2}+\frac{TnD+TnD}{2n})\\ \;\;\;\;=Oh(1+nD+T\cdot FV\cdot n+2TnD+TD) \end{array}$$

## 3. Numerical Experiments and Analysis

In this section, the performance of the proposed HAHA is simulated on 25 benchmark functions and CEC 2022 benchmark functions and compared with other optimization algorithms and other improved AHAs. The optimization ability, convergence, and statistical tests of HAHA are evaluated, which further verifies the superiority of HAHA in a series of evaluation indicators. In addition, in order to guarantee the reliability and persuasiveness of experimental results, the compilation environment for all experiments is the same; they are compiled and run on MATLABR2017b on Windows 11, AMD Ryzen 7 4800H with Radeon Graphics@2.90GHz, and 16GB RAM, and the experimental results are obtained by running each test function 30 times independently.

### 3.1. Benchmark Functions

As part of the study, 25 benchmark test functions and the challenging CEC 2022 test suite were used to evaluate the performance of the proposed HAHA. The details of the 25 benchmark functions used to verify the performance of the proposed HAHA are shown in Appendix A, Table A1. These functions contain uni-modal, multi-modal, hybrid, composition, and fixed-dimensional functions, which have good representation in evaluating algorithm performance. Among the 25 benchmark functions, F1 is a uni-modal function with a local extreme value, which is suitable for testing the utilization and local exploration abilities of the algorithm. F2–F5 are multi-modal functions with multiple local minima, which are usually used to evaluate the ability of algorithms to explore and jump out of local optima. F6–F10 are hybrid functions composed of multi-modal or uni-modal functions to test the balance between algorithm exploration and exploitation. Composite functions F11–F15 are often composed of basic functions and mixed functions, and the problem is more complicated. F16–F20 are fixed-dimensional functions taken from CEC 2019 test functions [71], and the complexity of the search space is significantly increased and more challenging, which is used to evaluate the comprehensive ability of the algorithm.

### 3.2. Algorithm Parameter Settings

The proposed HAHA is compared with other algorithms such as the original AHA [64], PSO [47], WOA [53], SCA [38], HHO [54], Seagull Optimization Algorithm (SOA) [57], SSA [58], AVOA [60], CryStAl [40], DMOA [62], Sand Cat Swarm Optimization (SCSO) [72], GJO [63], and AOAHA [65] to conduct comparative experiments to evaluate the performance of HAHA, where AOHAH is an improved algorithm of AHA. Table 2 shows the parameter settings of some algorithms, and the parameters of the rest of the comparison algorithms are the same as the corresponding references. Each algorithm is independently run 30 times on each benchmark function, and the calculation results of all algorithms are based on the average performance of the algorithms.

### 3.3. Results and Analyses for 25 Benchmark Functions 

In this experiment, in order to objectively and fairly evaluate the proposed HAHA, the average value (Avg) and standard deviation (Std) of the best solution obtained by independent running of each test function are compared as evaluation indicators, and the calculation formula is as follows [73]:

(18) $$\begin{array}{c} Avg=\frac{1}{runs}\sum_{i=1}^{runs}f_{i}^{*},\\ Std=\sqrt{\frac{1}{runs-1}\sum_{i=1}^{runs}{\left(f_{i}^{*}-Avg\right)}^{2}}, \end{array}$$

where $f_{i}^{*}$ is the best solution obtained in the *i*-th independent run, and *runs* represents the number of independent runs of the function.

Table 3 shows the statistical results of 14 algorithms running independently 30 times on 25 test functions, including Avg, Std, Wilcoxon rank-sum test *p*-value, and average rank. The best results are marked in bold. The uni-modal function F1 only has a global optimal value, which is used to test the local exploitation ability of the algorithm. The average value of the optimal solution obtained by HAHA on F1 is the smallest, which indicates that the proposed HAHA has very effective exploitation and utilization abilities. F2–F5 in Table 3 are the evaluation results of multi-modal functions. It can be seen that the experimental results of HAHA are basically better than competing algorithms on such functions, especially in solving F3 and F4 optimization problems, which proves that the proposed HAHA has good exploration ability and effectively avoids the algorithm from falling into the local optimal state.

Hybrid functions and combination functions are mostly used to evaluate the balance between algorithm development and exploration. As shown in Table 3, the proposed HAHA performance is often more competitive in functions F6, F7, F10, F12, and F15. Compared with other competitive algorithms, HAHA has a good balance between exploitation and exploration. F16–F20 are the fixed dimensions selected from CEC 2019, which are more challenging. F16 reaches the optimal value of 1 and has obvious advantages in F19, F21, F22, and F25, which further indicates that HAHA can better explore the optimal solution of complex problems. From the standard deviation, it can be seen that the performance of the proposed HAHA is stable. At the end of Table 3, the final ranking of each algorithm on 25 test sets is given, with HAHA ranking first on average.

In addition to statistical analysis of data by means and standard deviation, the Wilcoxon rank-sum test was also used to assess statistical differences between the proposed HAHA and other competing algorithms [74]. The *p*-value is used to determine whether the given algorithm is significantly different from the algorithm to be tested. When the *p*-value < 0.05, it indicates that the algorithm is significantly different from HAHA and has statistical significance. Table 3 presents the *p*-value for HAHA and other comparison algorithms for 25 benchmark functions. In most cases, the *p*-value of most algorithms is less than 0.05, indicating a significant difference between HAHA and other algorithms. The last line of Table 3 gives the number of significant differences, expressed by (+/=/−). Among them, “+” indicates that the algorithm being compared performs better than the proposed HAHA, “=” indicates that HAHA and this comparison algorithm have similar performance, and “−” indicates that the algorithm being compared is not as good as HAHA. Compared with the original AHA, HAHA and AHA have significant differences in 19 test functions, and HAHA’s performance is better than that of AHA. The HAHA has significant differences compared with PSO, WOA, SCA, HHO, SOA, SSA, AVOA, CryStAl, DMOA, SCSO, and GJO. Therefore, HAHA has good performance and is statistically significant.

Meanwhile, the Friedman test [75] is also commonly used as a popular method for non-parametric testing, and Table 4 shows the results of the Friedman test for each algorithm on 25 test functions. The proposed HAHA has the best Friedman test results for most test functions, and the average ranking of algorithm performance is shown in Table 4. Compared with other comparison algorithms, HAHA has the best average Friedman test result of 2.4 for 25 benchmark functions.

**Figure 4:**
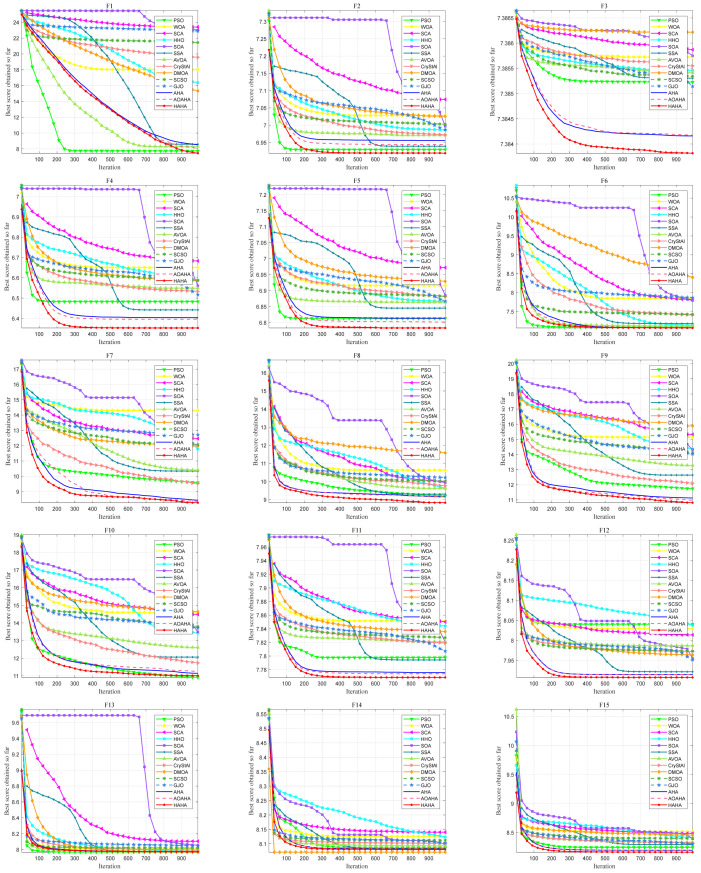

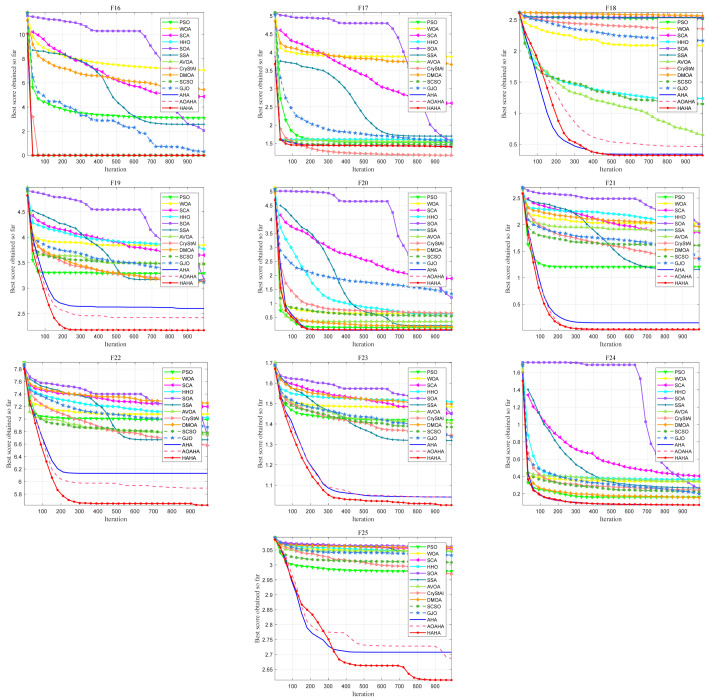
Convergence curves of the HAHA with other algorithms for 25 benchmark functions.

The convergence of the proposed algorithm is verified by comparing the convergence curves between the proposed HAHA and the competing algorithms. Figure 4 shows the convergence curves on 25 test functions, which are obtained by averaging the best solutions of the algorithm through 1000 iterations. As can be seen from Figure 4, the proposed HAHA is more competitive than the competitive algorithm. In the initial stage of iteration, HAHA converges faster. At the initial stage of iteration, F3, F7, F16, F19, F21, and F22 converge faster. With an increase in the number of iterations, the algorithm converges quickly to the optimal solution with high convergence accuracy. During the entire iteration process, the proposed HAHA maintains an intelligent balance between exploration and exploitation, effectively reducing the possibility of premature convergence of the algorithm.

**Figure 5:**
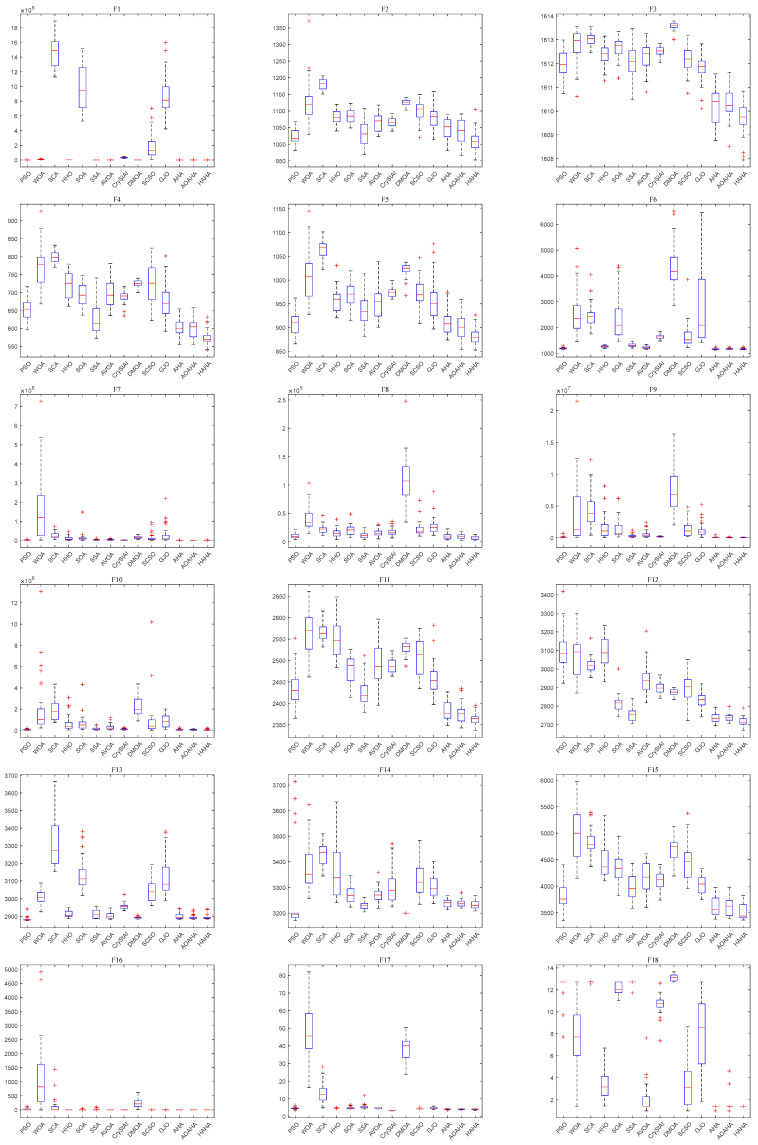

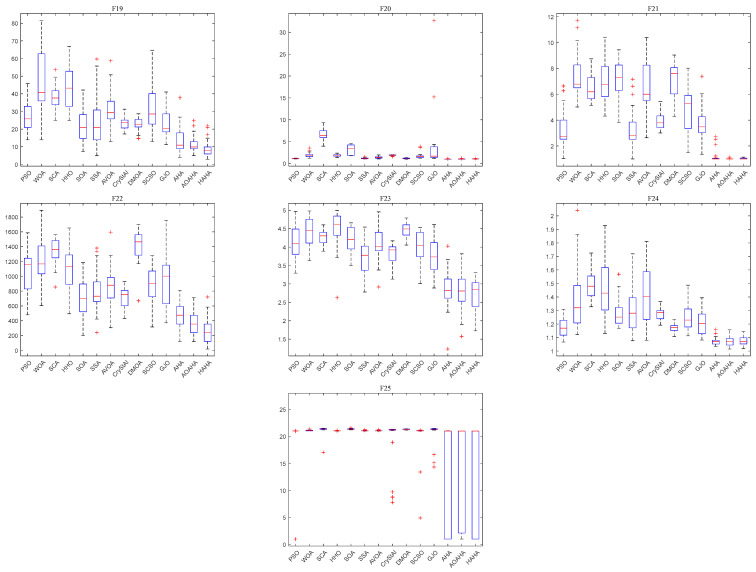
The box plots of the HAHA with other algorithms for 25 benchmark functions. "+" represents the frequency of abnormal situations, "−" represents the median line of the data.

**Figure 6:**
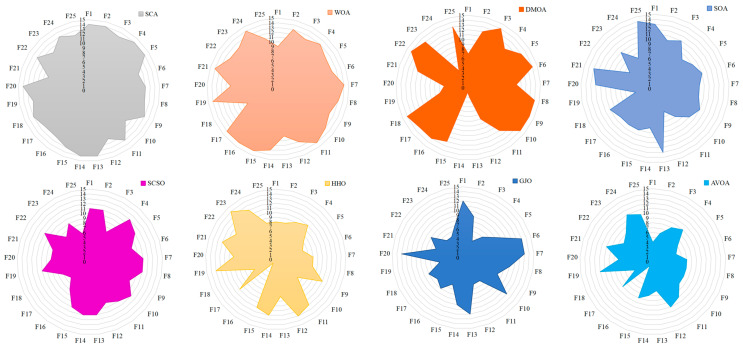

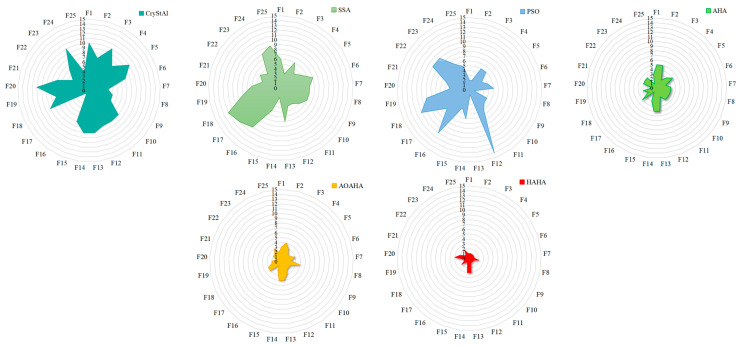
Radar charts of the proposed HAHA and other algorithms for 25 benchmark functions.

**Figure 7:**
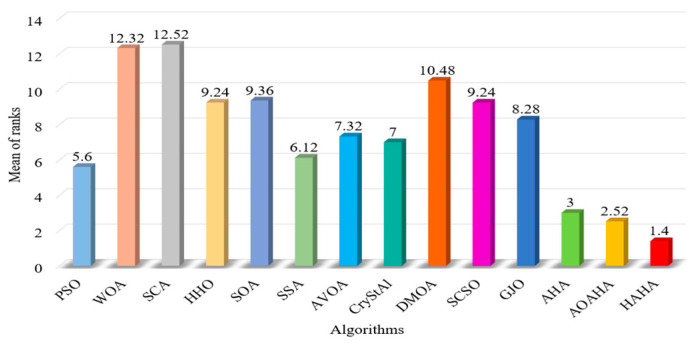
The average ranking of algorithms based on 25 benchmark functions.

Figure 5 shows box plots of the optimal solution distribution of each function. For most functions, the box graph position of HAHA is lower, indicating that the proposed HAHA has better performance and stronger robustness. Figure 6 shows the radar charts of comparison between HAHA and other competitive algorithms. The larger the area, the lower the ranking of the algorithm. On the contrary, the minimum area means that the overall performance of the algorithm is the best. Figure 7 shows that the average rank of the HAHA algorithm is the smallest, indicating that it ranks first among other competitive algorithms. This result proves the superiority of the proposed HAHA again.

The computational cost is also an important criterion for evaluating the performance of the algorithm. Table 5 shows the average runtime of HAHA and other competing algorithms in seconds on the test set. Compared with the original AHA, the calculation cost of HAHA is also inevitably increased, which is consistent with the previous analysis of calculation complexity and is a noticeable issue in the subsequent research.

To sum up, compared with other intelligent algorithms, HAHA effectively improves the exploration and development capabilities of the algorithm, avoids falling into local optima, and shows good competitiveness.

### 3.4. Results and Analyses on CEC 2022 Benchmark Functions

In this section, the latest CEC 2022 test functions are selected to further evaluate the performance of the proposed HAHA and compare it with other advanced intelligent algorithms and improved algorithms of AHA, including PSO [47], WOA [53], SCA [38], HHO [54], SOA [71], SSA [58], SAO [41], POA [62], Kepler Optimization Algorithm (KOA) [76], SCSO [72], GJO [63], AOAHA [65], and AHA [64]. The CEC 2022 test function simulates highly complex problems in global optimization, which is very challenging. In order to ensure the fairness and persuasiveness of the experimental results, all functions were tested in 10-dimensional space. The algorithm parameter settings were the same as in Table 2, and the experimental results were taken as an average of 30 independent runs.

**Figure 8:**
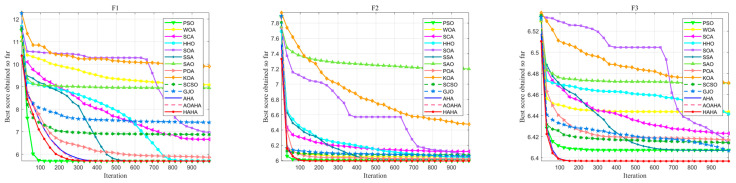

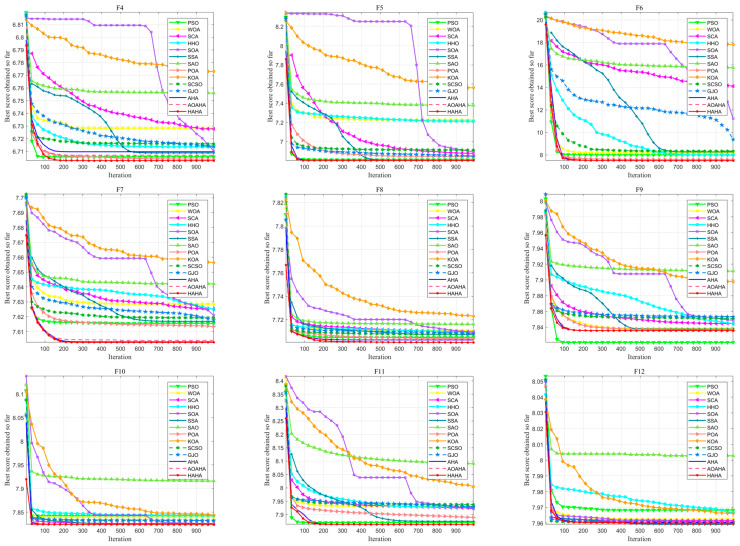
Convergence curves of the HAHA with other algorithms for CEC 2022 test functions.

Table 6 presents the experimental results of 30 independent runs of HAHA and other competitive algorithms on the CEC 2022 test set, including the mean, standard deviation, ranking, and *p*-value. This table demonstrates the best performance of HAHA among the 9 test functions, and the average ranking of HAHA is 1.250, which is the highest overall performance ranking. Using the Wilcoxon rank-sum test *p*-value to test whether there is a significant difference between HAHA and other algorithms, it can be seen from Table 6 that most algorithms have *p*-values less than 0.05, indicating that HAHA has statistical significance. From the convergence curves shown in Figure 8, it can be seen that the HAHA can effectively jump out of local optima and quickly approach the global optimal solution. The research results indicate that HAHA exhibits strong competitiveness and can serve as a powerful tool for solving global optimization problems.

## 4. Construction of CSGC–Ball Curves

In this section, first, the CSGC–Ball curves are defined, which are composed of *N*-segmented SGC–Ball curves and can construct more flexible and controllable complex curves. Secondly, in order to make the constructed curves smooth and continuous, the continuity conditions satisfying the G^1^ and G^2^ smooth splicing of the CSGC–Ball curves are studied, respectively. Finally, an example of CSGC–Ball curves is given.

**Definition 1.**  *The shape-adjustable generalized cubic Ball (SGC–Ball, for short) curves can be defined as [21]*

(19) $$P(t;\Omega )=\sum_{i=0}^{3}P_{i}b_{i,3}(t),t\in [0,1],$$

where $P_{i}\in R^{u}(u=2,3;i=0,1,2,3)$ are the control points of the curves; $\Omega =\{\omega ,\lambda_{1},\lambda_{2},\lambda_{3}\}$ are the shape parameters; ω∈[0,1] is the global shape parameter; and $\lambda_{1},\lambda_{3}\in [-3,3],\;\lambda_{2}\in [0,4]$ are the local shape parameters; and $b_{i,3}(t)(i=0,1,2,3)$ are the SCG–Ball curves basis functions defined as

(20) $$\left\{\begin{array}{l} b_{0,3}(t)=[1+(\lambda_{1}-1)\omega t(1-t)]{\left(1-t\right)}^{2},\\ b_{1,3}(t)=[2+\omega (1-\lambda_{1})+\omega (\lambda_{1}+\lambda_{2}-3)t]t{\left(1-t\right)}^{2},\\ b_{2,3}(t)=[2+\omega (2-\lambda_{2})-\omega (\lambda_{3}-\lambda_{2}+1)t]t^{2}(1-t),\\ b_{3,3}(t)=[1+(\lambda_{3}-1)\omega t(1-t)]t^{2}. \end{array}\right.$$

Compared with the traditional generalized Ball curves, the SGC–Ball curves have a satisfactory effect when constructing simple curve shapes, but for the construction of complex geometric curves in real life, the original single SGC–Ball curve is difficult to meet people’s requirements for curve construction and has certain limitations. Therefore, it is of great significance to construct the complex combination of SGC–Ball curves, which are defined as follows.

**Definition 2.**  *Given N + 1 nodes* $u_{0}<u_{1}<u_{2}<\cdots <u_{i}<u_{i+1}<\cdots <u_{N-1}<u_{N}$ *, the composite shape-adjustable generalized cubic Ball (abbreviation, CSGC–Ball) curves are defined as*

(21) $$\tilde{P}(u,\Omega ):=P_{j}\left(\frac{u-u_{j-1}}{h_{j}},\Omega_{j}\right),\;u\in [u_{j-1},u_{j}],j=1,2,\cdots ,N$$

where $h_{j}=u_{j}-u_{j-1}$, $\Omega_{j}=(\omega ,\lambda_{1,j},\lambda_{2,j},\lambda_{3,j}),j\in (1,2,\cdots ,N)$ are the shape parameters of the *j*-th SGC–Ball curves; ω∈[0,1] is the global shape parameter; and $\lambda_{1,j},\;\lambda_{3,j}\in [-3,3],\;$$\lambda_{2,j}\in [0,4],$ j∈(1,2,…,N) are local shape parameters of the CSGC–Ball curves. Meanwhile, the CSGC–Ball curves can be written as:

(22) $$\tilde{\Pi }:P_{j}(\frac{u-u_{j-1}}{h_{j}},\Omega_{j})=\sum_{i=0}^{3}P_{i,j}b_{i,3}(\frac{u-u_{j-1}}{h_{j}}),$$

where $P_{i,j}(i=0,1,2,3;j=1,2,\ldots ,N)$ represents the *i*-th control vertex of the *j*-th SGC–Ball curves.

The CSGC–Ball curve requires two adjacent curves to be smooth and continuous. In order to make the constructed CSGC–Ball curves meaningful, the G^1^ and G^2^ smooth continuity splicing conditions of the *j*-th and *j* + 1-th SGC–Ball curves are discussed below.

**Theorem 1.**  *If the control vertices and shape parameters of the j-segment and j + 1-segment SGC–Ball curves at node u_j_ satisfy*

(23) $$\left\{\begin{array}{l} P_{0,j+1}=P_{3,j},\\ P_{1,j+1}=\frac{kh_{j+1}(2-\omega (\lambda_{3,j}-1))}{h_{j}(2-\omega (\lambda_{1,j+1}-1))}(P_{3,j}-P_{2,j})+P_{0,j+1}. \end{array}\right.$$

then the CSGC–Ball curves are said to be G^1^ continuous at node *u_j_*. Among them, k>0. If the CSGC–Ball curves satisfy Equation (23) at each node *u_j_* (*j* = 1,…,*N*), then the CSGC–Ball curves are G^1^ continuous on the whole. In particular, when *k* = 1, Equation (23) is a necessary and sufficient condition for the CSGC–Ball curves to satisfy G^0^ continuity at nodes *u_j_* (*j* = 1,2…,*N*). The proof of Theorem 1 is given in Appendix B.1.

**Theorem 2.**  *If the control vertices and shape parameters of the j-segment and j + 1-segment SGC–Ball curves at node u_j_ satisfy*

(24) $$\left\{ \begin{array}{l} P_{0,j+1}=P_{3,j}\\ P_{1,j+1}=\frac{kh_{j+1}(2-\omega (\lambda_{3,j}-1))}{\;h_{j+1}(2-\omega (\lambda_{1,j+1}-1))}(P_{3,j}-P_{2,j})+P_{0,j+1}\\ P_{2,j+1}=\frac{2k^{2}h_{j+1}^{2}(3\omega (\lambda_{3,j}-1)-1)-\beta h_{j}h_{j+1}^{2}(2-\omega (\lambda_{3,j}-1))}{\;2h_{j}^{2}(\omega (\lambda_{2,j+1}-2)-2)}P_{3,j}\\ \;\;\;+\frac{-2k^{2}h_{j+1}^{2}(\omega (3\lambda_{3,j}-\lambda_{2,j}-1)-4)+\beta h_{j}h_{j+1}^{2}(2-\omega (\lambda_{3,j}-1))}{\;2h_{j}^{2}(\omega (\lambda_{2,j+1}-2)-2)}P_{2,j}\\ \;\;\;-\frac{k^{2}h_{j+1}^{2}(\omega (\lambda_{2,j}-2)+2)}{\;h_{j}^{2}(\omega (\lambda_{2,j+1}-2)-2)}P_{1,j}-\frac{k^{2}h_{j+1}^{2}}{\;h_{j}^{2}(\omega (\lambda_{2,j+1}-2)-2)}P_{0,j}\\ \;\;\;-\frac{\left(3\omega (\lambda_{1,j+1}-1)-1\right)}{\;\omega (\lambda_{2,j+1}-2)-2}P_{0,j+1}+\frac{\omega (3\lambda_{1,j+1}+\lambda_{2,j+1}-5)-4}{\;\omega (\lambda_{2,j+1}-2)-2}P_{1,j+1}+\frac{1}{\;\omega (\lambda_{2,j+1}-2)-2}P_{3,j+1}. \end{array}\right.$$

then the CSGC–Ball curves are said to be G^2^ continuous at connection note *u_j_*. Among them, k>0,β is an arbitrary constant. If the CSGC–Ball curves satisfy the G^2^ continuity condition at each node *u_j_* (*j* = 1,2,…,*N*), then the overall CSGC–Ball curves are G^2^ continuous. The proof of Theorem 2 is given in Appendix B.2.

**Figure 9:**
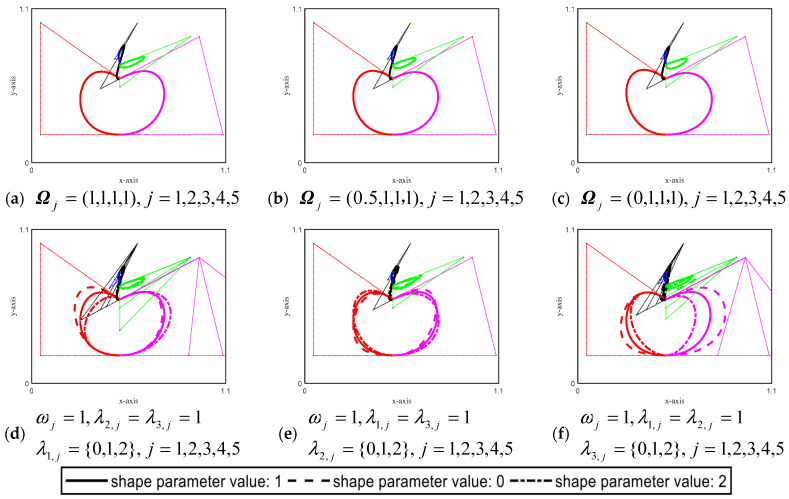
G^1^ splicing of the CSGC–Ball curves.

According to the CSGC–Ball curves definition and the G^1^ smooth splicing continuity condition of Theorem 1, Figure 9 gives examples of the CSGC–Ball curves graphs that satisfy the overall G^1^ smooth splicing condition when *N* = 5. Different colors represent each SGC–Ball curves to be spliced. $\Omega_{j}=(\omega ,\lambda_{1,j},\lambda_{2,j},\lambda_{3,j}),j\in (1,2,\ldots ,5)$ are the shape parameters of the *j*-th SGC–Ball curves of the CSGC–Ball curves, where ω is the global shape parameter of CSGC–Ball curves, and $\lambda_{1,j},\lambda_{2,j},\lambda_{3,j}j=1,2,\ldots ,5$ are the local shape parameters. The figure involves 16 variables, including 1 global shape parameter and 15 local shape parameters. Figure 9a–c describes the CSGC–Ball curves of the whole G^1^ smooth splicing, and the parameter values are $\Omega_{j}=(1,1,1,1),j=1,2,\ldots ,5$, $\Omega_{j}=(0.5,1,1,1),j=1,.2,\ldots ,5$ and $\Omega_{j}=(0,1,1,1),j=1,2,\ldots ,5$, respectively. The local shape parameters are the same, but the overall shape parameter value is different, reflecting the whole G^1^ smooth splicing CSGC–Ball curve. It can be seen that ω controls the overall shape change of the graphs. Figure 9d–f discusses the comparison curves on the same shape with different local shape parameters. The solid line “-” represents the curves with the given local shape parameter value of 1, the dotted line “--” represents the curves with the given local shape parameter value taken as 0, and the dotted line “-.” represents the curves with the given local shape parameter value of 2. From Figure 9, it can be found that local shape parameters control the local shape changes of the CSGC–Ball curves, and the overall shape parameters control the overall shape of the CSGC–Ball curves. When different shape parameters change, the control points of the curves also change, and the curves are close to the corresponding control points.

**Figure 10:**
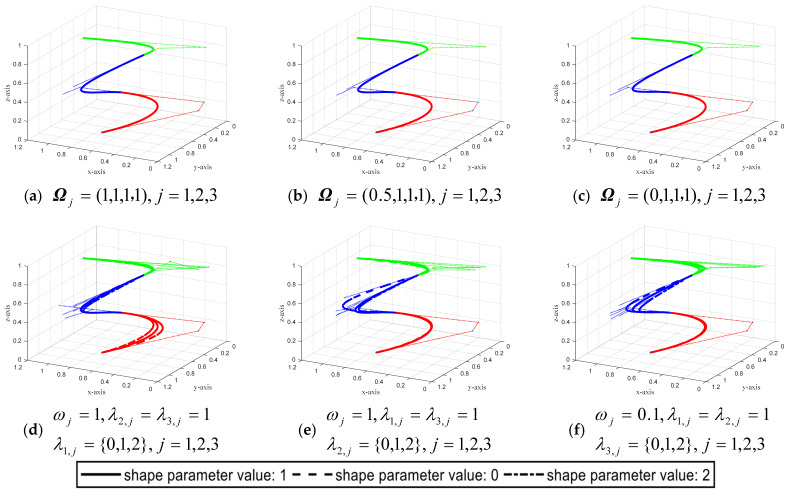
G^2^ splicing of the CSGC–Ball curves.

According to the G^2^ smooth splicing continuity condition of CSGC–Ball curves given by Theorem 2, Figure 10 shows examples of the spatial curve designed by CSGC–Ball curves of the overall G^2^ smooth splicing when *N* = 3. This CSGC–Ball curve involves 10 variables, including 1 global shape parameter and 9 local shape parameters. Figure 10a–c shows the whole G^2^ smooth CSGC–Ball curves of shape parameters are $\Omega_{j}=(1,1,1,1),j=1,2,3$, $\Omega_{j}=(0.5,1,1,1),j=1,2,3$ and $\Omega_{j}=(0,1,1,1),j=1,2,3$, respectively. Figure 10d–f displays the comparison curves on the same graph when the given local shape parameter values are different. When the shape parameters are different, the CSGC–Ball curves will appropriately change some control points of the curves to meet the overall G^2^ smooth stitching continuity condition.

## 5. Application of HAHA in CSGC–Ball Curve-Shape Optimization

### 5.1. CSGC–Ball Curve-Shape Optimization Model

The bending energy value of the curves can approximately reflect the smoothness of the curves, and they are negatively correlated when the bending energy value of the curves is smaller, the smoothness of the curves is better, and vice versa. Therefore, the G^1^ and G^2^ continuous shape optimization models of CSGC–Ball curves can be established, respectively, according to the minimum value of the curve bending energy.

Assuming that $E_{j}(\Omega_{j})$ represents the bending energy of the *j*-th SGC–Ball curve, the calculation formula of its energy is

(25) $$E_{j}(\Omega_{j})=\int_{0}^{1}\left|\right|P_{j}{}^{\prime \prime }(\frac{u-u_{j-1}}{h_{j}};\Omega_{j}){\left|\right|}^{2}dt\;\;\;\;\;\;\;u\in [u_{j-1},u_{j}]$$

where $h_{j}=u_{j}-u_{j-1}$, $\Omega_{j}=(\omega ,\lambda_{1,j},\lambda_{2,j},\lambda_{3,j}),j\in (1,2,\ldots ,N)$ are shape parameters of the *j*-segment SGC–Ball curve; ω∈[0,1] is the global shape parameter; and $\lambda_{1,j},\lambda_{3,j}\in [-3,3],\lambda_{2,j}\in [0,4],j\in (1,2,\ldots ,N)$ are local shape parameters.

Therefore, the bending energy *E* of the combined CSGC–Ball curves can be expressed by Equation (26):

(26) $$E=\sum_{j=1}^{n}E_{j}(\Omega_{j})=\sum_{j=1}^{n}\int_{0}^{1}\left|\right|P_{j}{}^{\prime \prime }(\frac{u-u_{j-1}}{h_{j}};\Omega_{j}){\left|\right|}^{2}dt$$

where $\Omega_{j}=\{\omega ,\lambda_{1,j},\lambda_{2,j},\lambda_{3,j}\}$ are the shape optimization parameters of the CSGC–Ball curves.

Through the energy function of CSGC–Ball curves, the energy minimum shape optimization model of CSGC–Ball curves is expressed by Equation (27):

(27) $$\underset{\Omega_{j}}{\arg\;\min\;}E=\sum_{j=1}^{n}E_{j}(\Omega_{j})=\sum_{j=1}^{n}\int_{0}^{1}\left|\right|P_{j}{}^{\prime \prime }(\frac{u-u_{j-1}}{h_{j}};\Omega_{j}){\left|\right|}^{2}dt$$

Substituting Equation (22) into Equation (27), the bending energy of the *j*-th segment of CSGC–Ball curves can be obtained through simple calculation, and its expression is shown in Equation (28):

(28) $$\begin{array}{l} \int_{0}^{1}||P_{j}{}^{\prime \prime }(\frac{u-u_{j-1}}{h_{j}};\Omega_{j})|{|}^{2}dt\\ =\int_{0}^{1}||\sum_{i=0}^{3}P_{i,j}b_{i,3}^{\prime \prime }(\frac{u-u_{j-1}}{h_{j}})|{|}^{2}dt\\ =a_{0,j}||P_{0,j}|{|}^{2}+a_{1,j}||P_{1,j}|{|}^{2}+a_{2,j}||P_{2,j}|{|}^{2}+a_{3,j}||P_{3,j}|{|}^{2}+2a_{4,j}P_{0,j}P_{1,j}+2a_{5,j}P_{0,j}P_{2,j}\\ \;\;+2a_{6,j}P_{0,j}P_{3,j}+2a_{7,j}P_{1,j}P_{2,j}+2a_{8,j}P_{1,j}P_{3,j}+2a_{9,j}P_{2,j}P_{3,j}. \end{array}$$

where

$$a_{0,j}={\left(6\omega (\lambda_{1,j}-1)-2\right)}^{2}+\frac{155}{5}\omega^{2}{\left(\lambda_{1,j}-1\right)}^{2}-10\omega (\lambda_{1,j}-1)(6\omega (\lambda_{1,j}-1)-2);$$

$$a_{1,j}=\frac{24}{5}\lambda_{1,j}^{2}\omega^{2}+\frac{8}{5}\lambda_{1,j}\lambda_{2,j}\omega^{2}-\frac{64}{5}\lambda_{1,j}\omega^{2}-16\lambda_{1,j}\omega +\frac{4}{5}\lambda_{2,j}^{2}\omega^{2}-\frac{24}{5}\lambda_{2,j}\omega^{2}+\frac{56}{5}\omega^{2}+16\omega +16;$$

$$a_{2,j}=\frac{4}{5}\lambda_{2,j}^{2}\omega^{2}+\frac{8}{5}\lambda_{2,j}\lambda_{3,j}\omega^{2}-\frac{8}{5}\lambda_{2,j}\omega^{2}+\frac{24}{5}\lambda_{3,j}^{2}\omega^{2}-\frac{32}{5}\lambda_{3,j}\omega^{2}-16\lambda_{3,j}\omega +\frac{24}{5}\omega^{2}+16\omega +16;$$

$$a_{3,j}=\frac{24}{5}\omega^{2}{\left(\lambda_{3,j}-1\right)}^{2}-4\omega (\lambda_{3,j}-1)+4;$$

$$a_{4,j}=10\lambda_{1,j}\omega -10\omega +\frac{56}{5}\lambda_{1,j}\omega^{2}+\frac{4}{5}\lambda_{2,j}\omega^{2}-\frac{32}{5}\omega^{2}-\frac{24}{5}\lambda_{1,j}^{2}\omega^{2}-\frac{4}{5}\lambda_{1,j}\lambda_{2,j}\omega^{2}-4;$$

$$a_{5,j}=(2+4\lambda_{1,j}+2\lambda_{3,j})\omega -(\frac{14}{5}\lambda_{1,j}+\frac{4}{5}\lambda_{2,j}+\frac{6}{5}\lambda_{3,j}-\frac{14}{5}-\frac{4}{5}\lambda_{1,j}\lambda_{2,j}-\frac{6}{5}\lambda_{2,j}\lambda_{3,j})\omega^{2}-4;$$

$$a_{6,j}=\omega_{j}(\lambda_{3,j}-1)(6\omega (\lambda_{1,j}-1)-2)-2\omega (\lambda_{1,j}-1)-\frac{36}{5}\omega^{2}(\lambda_{1,j}-1)(\lambda_{3,j}-1)+4;$$

$$\begin{array}{c} a_{7,j}=(4\lambda_{1,j}-8+4\lambda_{3,j})\omega +(\frac{14}{5}\lambda_{1,j}+\frac{16}{5}\lambda_{2,j}-\frac{2}{5}\lambda_{3,j}-\frac{22}{5}-\frac{4}{5}\lambda_{2,j}^{2}-\frac{4}{5}\lambda_{1,j}\lambda_{2,j}-\frac{6}{5}\lambda_{1,j}\lambda_{3,j}+\\ \frac{4}{5}\lambda_{2,j}\lambda_{3,j})\omega^{2}-8; \end{array}$$

$$a_{8,j}=(2+2\lambda_{1,j}-4\lambda_{3,j})\omega -(\frac{6}{5}\lambda_{1,j}-\frac{4}{5}\lambda_{2,j}+\frac{2}{5}\lambda_{3,j}+\frac{2}{5}-\frac{6}{5}\lambda_{1,j}\lambda_{3,j}+\frac{4}{5}\lambda_{2,j}\lambda_{3,j})\omega^{2}-4;$$

$$a_{9,j}=10\lambda_{3,j}\omega -10\omega -\frac{4}{5}\lambda_{2,j}\omega^{2}+8\lambda_{3,j}\omega^{2}-\frac{16}{5}\omega^{2}-\frac{24}{5}\lambda_{3,j}\omega^{2}+\frac{4}{5}\lambda_{2,j}\lambda_{3,j}\omega^{2}-4.$$

Combined with Equations (27) and (28), the shape optimization model of CSGC–Ball curves can be written as

(29) $$\begin{array}{c} \underset{\Omega_{j}}{\arg\;\min\;}E=\sum_{j=1}^{n}(a_{0,j}||P_{0,j}|{|}^{2}+a_{1,j}||P_{1,j}|{|}^{2}+a_{2,j}||P_{2,j}|{|}^{2}+a_{3,j}||P_{3,j}|{|}^{2}+2a_{4,j}P_{0,j}\cdot P_{1,j}+\\ \;\;\;\;\;\;\;\;2a_{5,j}P_{0,j}\cdot P_{2,j}+2a_{6,j}P_{0,j}\cdot P_{3,j}+2a_{7,j}P_{1,j}\cdot P_{2,j}+2a_{8,j}P_{1,j}\cdot P_{3,j}+2a_{9,j}P_{2,j}\cdot P_{3,j}), \end{array}$$

and the constraint conditions of the whole G^1^ and G^2^ continuous are Equation (23) and Equation (24), respectively.

Due to the high nonlinearity of the objective function, it is not an easy task to solve the established optimization model using traditional optimization methods. Therefore, the objective function of the CSGC–Ball curve-shape optimization models is regarded as the fitness function, and the proposed HAHA algorithm can be used to obtain the energy optimal solution of the established optimization models.

### 5.2. Steps for HAHA to Solve the CSGC–Ball Curve-Shape Optimization Model

This subsection will introduce the detailed steps to solve the established CSGC–Ball curve-shape optimization model with the proposed HAHA, which are described as follows:

**Step 1:** Set relevant parameters, for example, *n*, *T*, *Ub*, *Lb*, and the CSGC–Ball curve control points;

**Step 2:** Initialization. Randomly initialize the hummingbird population by Equation (1) when *t* = 1, obtain the positions of *n* hummingbirds, take the bending energy value *E* of the CSGC–Ball curves as the fitness function, calculate *E* of each individual, record the best fitness value as the problem optimal solution *E_best_*, and initialize the visit table;

**Step 3:** If *rand* > 0.5, perform Step 5 and Step 6; otherwise, use Equation (6) to obtain the candidate solution *v_i_* (*t* + 1) of guided foraging, and obtain the elite opposition-based solution *x_i_*_,*elite*_ (*t* + 1) by Equation (10), if *E* (*x_i_*_,*elite*_ (*t* + 1)) < *E* (*v_i_* (*t* + 1)), then *v_i_* (*t* + 1)= *x_i_*_,*elite*_ (*t* + 1);

**Step 4:** If *E* (*v_i_* (*t* + 1)) < *E* (*x_i_* (*t*)), then *x_i_* (*t* + 1) = *v_i_* (*t + 1*), and update the visit table;

**Step 5:** Use Equation (8) to execute the territory foraging strategy of hummingbirds to obtain candidate solutions *v_i_* (*t* + 1), and obtain solutions *v_i_*_,*p*_ (*t* + 1) by Equation (11), if *E*(*v_i_*_,*p*_ (*t* + 1)) < *E*(*v_i_* (*t* + 1)), then *v_i_* (*t + 1*) = *v_i_*_,*p*_ (*t* + 1),

**Step 6:** If *E* (*v_i_* (*t* + 1)) < *E* (*x_i_* (*t*)), then *x_i_* (*t + 1*) = *v_i_* (*t* + 1), and update the visit table;

**Step 7:** If mod(*t*, 2*n*) == 0, then the solution with the largest energy value is used for migration foraging by Equation (9) to obtain the random solution *x_wor_* (*t* + 1), and use Equation (16) to perform Cauchy mutation on it to obtain the mutated solution *x_cauchy_* (*t* + 1). If *E* (*x_cauchy_* (*t* + 1)) < *E* (*x_wor_* (*t* + 1)), then *x_wor_* (*t + 1*) = *x_cauchy_* (*t* + 1), update the visit table. Otherwise, perform Step 8.

**Step 8:** *t* = *t* + 1, if *t* < *T*, then return to Step 3, otherwise execute Step 9;

**Step 9:** Output the energy best value *E*_best_ of the established CSGC–Ball curves and the corresponding shape parameter values.

### 5.3. Numerical Examples

In order to demonstrate the effectiveness and excellence of the proposed HAHA in solving the established CSGC–Ball curve-shape optimization model. In this section, some representative numerical examples are given to solve the established optimization model using advanced algorithms such as HAHA, and the results are compared and studied. In all numerical examples, the algorithm parameters are shown in Table 2, the population size is 50, and the maximum number of iterations is 1000.

**Example 5.1** This numerical example graphically presents the “letter W” graph designed by the complex CSGC–Ball curves that satisfy the overall G^2^ smooth splicing continuity condition. The graph shape is composed of eight-segment SGC–Ball curves G^2^ smoothly spliced; different colors represent different SGC–Ball curves; the black lines are auxiliary lines. The convergence curves when the objective function of the optimization model converges to the optimal value are also provided. In this example, for the CSGC–Ball curves with overall G^2^ smooth splicing, it is only necessary to give the coordinates of control points $P_{0,0},P_{0,1},P_{0,2},P_{0,3},P_{1,3},P_{2,3},P_{3,3}$ and $P_{4,0},P_{4,1},P_{4,2},P_{4,3},P_{5,3},P_{6,3},P_{7,3}$. The remaining control vertices of the curves to be spliced can be calculated according to the G^2^ smooth continuity splicing condition and the coordinates of the known control vertices.

**Figure 11:**
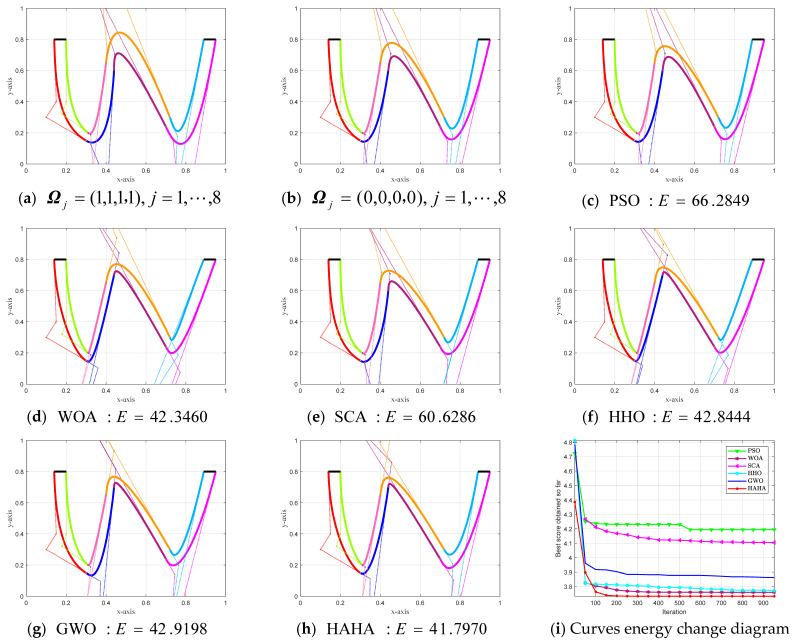
Shape optimization of the CSGC–Ball curves of overall G^2^ splicing.

In this example, there are a total of 25 shape parameters that need to be optimized, including 1 global parameter and 24 local shape parameters. Figure 11 shows the CSCG–Ball curves and the energy change convergence diagrams obtained by solving the established shape optimization model using optimization algorithms such as HAHA. Figure 11a,b shows the “letter W” shape CSCG–Ball curves of the overall G^2^ smooth splicing for given the shape parameter value. Figure 11c,h, respectively, describes the CSCG–Ball curves of smooth splicing of the overall G^2^ with minimum energy obtained after optimization by PSO, WOA, SCA, HHO, GWO, and HAHA. Figure 11i shows the energy change diagrams of each algorithm to solve the G^2^ smooth splicing shape optimization mode, and the proposed HAHA solves the model with the highest convergence accuracy.

Appendix C Table A2 shows the optimal shape parameters and minimum energy values obtained by the corresponding intelligent algorithm to solve the overall G^2^ smooth splicing shape optimization model. It proves that the proposed HAHA algorithm is more competitive and has advantages over other optimization algorithms in solving the optimization model of the CSCG–Ball curves that satisfy the G^2^ smooth splicing continuity condition. The final result has a minimum energy value of 41.7970 to obtain the smoothest graphics.

**Example 5.2** This example gives the “snail on grass” diagram designed by the complex CSCG–Ball curves of the hybrid smooth splicing of G^0^, G^1^, and G^2^ in graphical form, and the convergence curves of the optimization model converging to the optimal value are given. Different colors represent different curves, and the graph is composed of 29 SGC–Ball curves involving 88 shape optimization parameters, including 1 overall shape parameter and 87 local shape parameters. Using PSO, WOA, SCA, HHO, SMA (Slime Mould Algorithm) [77], and HAHA to solve the shape optimization model, the ideal optimal shape of the CSCG–Ball curves that satisfies the smooth splicing of mixed G^0^, G^1^, and G^2^ can be obtained.

**Figure 12:**
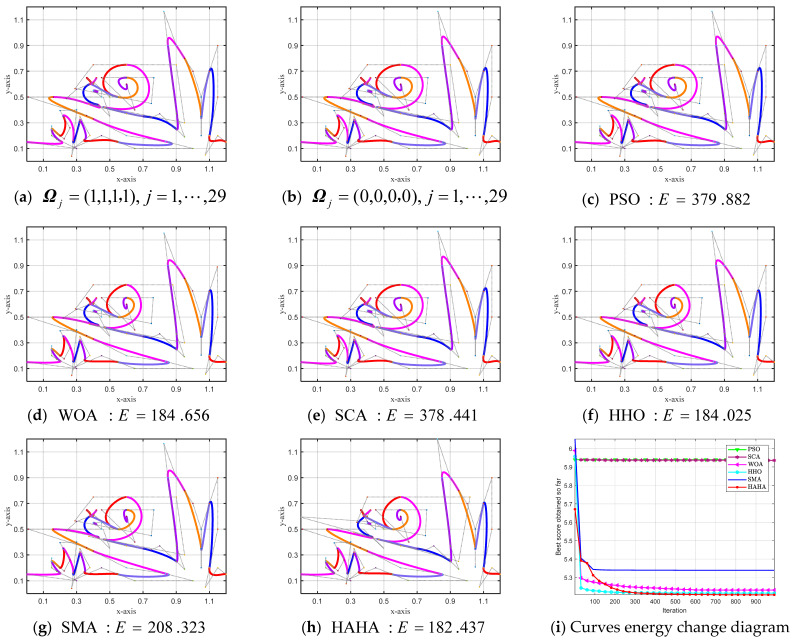
Shape optimization of CSGC–Ball curves for mixed G^0^, G^1^, and G^2^ splicing.

Figure 12 shows the CSCG–Ball curves and the energy change diagrams with the smooth splicing of the hybrid G^0^, G^1^, and G^2^ with the smallest energy obtained by solving the curve-shape optimization model established by the HAHA algorithm and other advanced optimization algorithms. Among them, Figure 12a,b is the graphs constructed from the CSCG–Ball curves that are blended G^0^, G^1^, and G^2^ spliced smoothly with freely given shape parameter values. Figure 12c–h shows the CSCG–Ball curves with smooth splicing of mixed G^0^, G^1^, and G^2^ with minimum energy obtained after optimization by different optimization algorithms, respectively. Figure 12i shows the energy change diagram of each algorithm to solve the hybrid G^0^, G^1^, and G^2^ smooth splicing shape optimization models. When the number of iterations reaches 200, the energy value of the model solved by HAHA tends to be stable, and compared with other algorithms, HAHA has the highest convergence accuracy.

Appendix D Table A3 shows the optimal shape parameter values and the minimum energy values of the graphs designed by the mixed G^0^, G^1^, and G^2^ smoothly spliced CSCG–Ball curves obtained by each algorithm. Among all the algorithms, the CSGC–Ball curve obtained by the proposed HAHA with the smooth splicing of mixed G^0^, G^1^, and G^2^ is the smoothest, and the obtained energy value is 182.437. The effectiveness of HAHA in solving the CSGC–Ball curve-shape optimization model is fully demonstrated.

## 6. Conclusions and Future Research

In this paper, complex CSCG–Ball curves with global and local shape parameters are constructed based on SGC–Ball basis functions, and the geometric conditions for G^1^ and G^2^ continuity splicing between adjacent SCG–Ball curves are studied. The constructed CSGC–Ball curves can not only construct more complex geometric product shapes in reality but also adjust the overall or local shape of the curves more flexibly by changing the overall or local shape parameters, thereby making the curves have higher shape adjustability.

In addition, a novel improved HAHA is proposed, which combines EOL, PSO, and Cauchy mutations with AHA. The introduction of the EOL strategy better balances the exploration and exploitation of algorithms and increases their optimization ability. In the exploitation stage, combined with the PSO strategy, the convergence speed is accelerated and the optimization ability of the algorithm is improved. Cauchy mutations are added to increase the diversity of the population and improve the ability of the algorithm to jump out of the local optimal. In order to better evaluate the overall performance of HAHA compared with other advanced intelligent algorithms for 25 benchmark functions and the CEC 2022 test set, the experimental results verify that the proposed HAHA has certain superiority and competitiveness in solving global optimization problems.

Finally, according to the minimum bending energy of the curves, the CSGC–Ball curve-shape optimization models are established, and the specific steps for HAHA to solve the CSGC–Ball shape optimization model are given. Two representative numerical examples verify the effectiveness of HAHA in solving the CSGC–Ball curve-shape optimization models. However, it is worth noting that the HAHA proposed in this paper exhibits advantages and competitiveness in solving optimization problems with continuous variables, but there are certain limitations in solving problems in non-continuous decision spaces. In future research, the proposed HAHA can be used to solve optimization problems in the fields of feature selection, image segmentation, and machine learning. In addition, we will consider extending the research technique of combined SGC–Ball interpolation curves to the CQGS–Ball surfaces in [78] and utilizing intelligent algorithms in [79,80,81] to investigate the shape optimization problem of the surfaces.

## Figures and Tables

**Table 2:** Parameter settings of each algorithm.

Algorithm	Parameters	Setting Value
All algorithm	Population size (*n*)	100
	Max iterations (*T*)	1000
	Number of runs	30
AHA	Migration coefficient (*M*)	2*n*
HAHA	Learning factors (*c*_1_, *c*_2_)	2
	Migration coefficient (*M*)	2*n*
PSO	Neighboring ratio	0.25
	Inertia weight (ω)	0.9
	Cognitive and social factors	*c*_1_ = *c*_2_ = 1.5
WOA	Parameter (*a*)	from 2 to 0
SCA	Constant (*a*)	2
HHO	Energy (*E*_1_)	from 2 to 0
SOA	Control factor (*f_c_*)	2
AOA	C_3_, C_4_	C_3_ = 1,C_4_ = 2
GJO	Decreasing energy of the prey (E1)	from 1.5 to 0
	Constant values (β, c_1_)	1.5, 1.5
POA	R	0.2
SCSO	Sensitivity range (*r_G_*)	from 2 to 0
	R	from −2*r_G_* to 2*r_G_*
KOA	$\bar{T}$, *μ*_0_, *γ*	3, 0.1, 15

**Table 3:** The comparison results of HAHA and other algorithms for 25 benchmark functions.

F	Index	Algorithms													
		PSO	WOA	SCA	HHO	SOA	SSA	AVOA	CryStAl	DMOA	SCSO	GJO	AHA	AOAHA	HAHA
F1	Avg.	2.29 × 10^3^	6.75 × 10^7^	1.46 × 10^10^	1.21 × 10^7^	9.84 × 10^9^	5.35 × 10³	4.04 × 10³	3.11 × 10^8^	4.40 × 10^6^	2.05 × 10^9^	8.79 × 10^9^	4.87 × 10³	3.12 × 10³	**1.64 ×** **10^3^**
	Std.	3.60 × 10³	3.95 × 10^7^	2.15 × 10^9^	2.42 × 10^6^	2.89 × 10^9^	5.89 × 10³	4.80 × 10³	7.57 × 10^7^	2.91 × 10^6^	1.87 × 10^9^	2.68 × 10^9^	6.17 × 10³	3.58 × 10³	1.94 × 10³
	*p*-value	0.185767	3.02 × 10^−11^	3.02 × 10^−11^	3.02 × 10^−11^	3.02 × 10^−11^	3.67 × 10^−3^	1.49 × 10^−1^	3.02 × 10^−11^	3.02 × 10^−11^	3.02 × 10^−11^	3.02 × 10^−11^	0.001370	0.036439	\
	Rank	2	9	14	8	13	6	4	10	7	11	12	5	3	1
F2	Avg.	1.02 × 10³	1.13 × 10³	1.18 × 10³	1.08 × 10³	1.08 × 10³	1.03 × 10³	1.07 × 10³	1.07 × 10³	1.12 × 10³	1.10 × 10³	1.08 × 10³	1.05 × 10³	1.04 × 10³	**1.01 × 10³**
	Std.	24.2	65.4	16.5	20.6	22	38.6	27.9	12.4	10.5	30.3	37	29.2	37.6	35.3
	*p*-value	0.133454	4.20 × 10^−10^	3.02 × 10^−11^	2.67 × 10^−9^	1.69 × 10^−9^	5.55 × 10^−2^	1.60 × 10^−7^	1.56 × 10^−8^	3.69 × 10^−11^	1.07 × 10^−9^	5.53 × 10^−8^	6.36 × 10^−5^	0.009468	\
	Rank	2	13	14	8	10	3	6	7	12	11	9	5	4	1
F3	Avg.	1611.994	1612.794	1613.027	1612.368	1612.638	1612.121	1612.296	1612.512	1613.589	1612.173	1611.799	1610.274	1610.294	**1609.722**
	Std.	0.572	0.677	0.248	0.483	0.515	0.585	0.564	0.205	0.152	0.554	0.599	0.7471	0.624	0.710
	*p*-value	3.34 × 10^−11^	3.69 × 10^−11^	3.02 × 10^−11^	3.02 × 10^−11^	3.02 × 10^−11^	4.50 × 10^−11^	3.34 × 10^−11^	3.02 × 10^−11^	3.02 × 10^−11^	3.34 × 10^−11^	9.92 × 10^−11^	0.011711	0.001236	\
	Rank	5	12	13	9	11	6	8	10	14	7	4	2	3	1
F4	Avg.	653.6209	773.0286	798.6650	723.8702	694.3867	627.5682	698.6022	686.8479	725.0058	726.1686	674.7303	604.0870	599.1974	**574.0913**
	Std.	28.5	60.2	17.3	37.6	31.6	44.8	38.2	16.9	9.40	50.7	47.7	24.1	24.4	22.6
	*p*-value	1.96 × 10^−10^	3.02 × 10^−11^	3.02 × 10^−11^	3.02 × 10^−11^	3.02 × 10^−11^	2.38 × 10^−7^	3.02 × 10^−11^	3.02 × 10^−11^	3.02 × 10^−11^	3.34 × 10^−11^	1.33 × 10^−10^	1.09 × 10^−5^	2.53 × 10^−4^	\
	Rank	5	13	14	10	8	4	9	7	11	12	6	3	2	1
F5	Avg.	909.8099	1008.153	1066.307	957.5451	969.0228	939.2126	956.6075	973.4062	1020.7300	974.4398	959.1655	909.8768	899.4413	**882.7806**
	Std.	23.6	49.1	17.8	23.2	26.1	34.9	34.8	9.56	14.7	29.1	46.3	24.4	25	17.4
	*p*-value	2.00 × 10^−5^	3.02 × 10^−11^	3.02 × 10^−11^	3.69 × 10^−11^	4.08 × 10^−11^	3.20 × 10^−9^	1.96 × 10^−10^	3.02 × 10^−11^	3.02 × 10^−11^	4.08 × 10^−11^	2.61 × 10^−10^	1.17 × 10^−5^	5.57 × 10^−3^	\
	Rank	3	12	14	7	9	5	6	10	13	11	8	4	2	1
F6	Avg.	1195.395	2558.867	2492.894	1259.143	2400.151	1314.833	1234.539	1643.442	4375.550	1675.289	2608.576	1171.708	1172.995	**1168.996**
	Std.	30.3	908	503	39.5	886	64.8	52.7	89.6	833	509	1.31 × 10^3^	29.6	23.6	32.7
	*p*-value	9.03 × 10^−4^	3.02 × 10^−11^	3.02 × 10^−11^	1.29 × 10^−9^	3.02 × 10^−11^	1.61 × 10^−10^	4.44 × 10^−7^	3.02 × 10^−11^	3.02 × 10^−11^	4.50 × 10^−11^	3.02 × 10^−11^	0.510598	0.157976	\
	Rank	4	12	11	6	10	7	5	8	14	9	13	2	3	1
F7	Avg.	1.45 × 10^4^	1.62 × 10^6^	2.58 × 10^5^	1.12 × 10^5^	1.54 × 10^5^	3.09 × 10^4^	3.32 × 10^4^	1.35 × 10^4^	1.59 × 10^5^	1.77 × 10^5^	3.32 × 10^5^	4.61 × 10^3^	4.30 × 10^3^	**3.92 × 10^3^**
	Std.	1.41 × 10^4^	1.77 × 10^6^	1.55 × 10^5^	1.18 × 10^5^	2.59 × 10^5^	2.64 × 10^4^	2.88 × 10^4^	7.59 × 10^3^	7.42 × 10^4^	2.67 × 10^5^	5.04 × 10^5^	4.01 × 10^3^	3.16 × 10^3^	5.08 × 10^3^
	*p*-value	2.00 × 10^−6^	3.69 × 10^−11^	3.02 × 10^−11^	1.09 × 10^−10^	6.07 × 10^−11^	5.57 × 10^−10^	9.76 × 10^−10^	2.39 × 10^−8^	3.02 × 10^−11^	7.39 × 10^−11^	3.69 × 10^−11^	0.045146	0.000201	\
	Rank	5	14	12	8	9	6	7	4	10	11	13	3	2	1
F8	Avg.	**5.43 × 10^4^**	2.15 × 10^6^	1.94 × 10^6^	6.56 × 10^5^	6.74 × 10^5^	1.73 × 10^5^	3.00 × 10^5^	1.23 × 10^5^	2.25 × 10^6^	9.78 × 10^5^	8.99 × 10^5^	6.87 × 10^4^	7.70 × 10^4^	6.03 × 10^4^
	Std.	5.07 × 10^4^	2.80 × 10^6^	9.57 × 10^5^	7.41 × 10^5^	7.98 × 10^5^	1.36 × 10^5^	2.83 × 10^5^	5.86 × 10^4^	9.58 × 10^5^	1.98 × 10^6^	6.08 × 10^5^	5.96 × 10^4^	5.19 × 10^4^	5.47 × 10^4^
	*p*-value	0.348	3.34 × 10^−11^	3.02 × 10^−11^	6.12 × 10^−10^	5.07 × 10^−10^	3.83 × 10^−5^	1.25 × 10^−7^	1.17 × 10^−5^	3.02 × 10^−11^	5.97 × 10^−9^	8.99 × 10^−11^	0.003501	0.141277	\
	Rank	1	13	12	8	9	6	7	5	14	11	10	3	4	2
F9	Avg.	1.25 × 10^5^	3.76 × 10^6^	4.54 × 10^6^	1.67 × 10^6^	1.47 × 10^6^	3.07 × 10^5^	5.83 × 10^5^	1.80 × 10^5^	7.66 × 10^6^	1.37 × 10^6^	1.23 × 10^6^	6.74 × 10^4^	5.69 × 10^4^	**4.90 × 10^4^**
	Std.	1.19 × 10^5^	4.88 × 10^6^	2.83 × 10^6^	1.87 × 10^6^	1.61 × 10^6^	2.53 × 10^5^	6.29 × 10^5^	6.22 × 10^4^	3.64 × 10^6^	1.24 × 10^6^	1.16 × 10^6^	7.94 × 10^4^	3.58 × 10^4^	3.21 × 10^4^
	*p*-value	1.11 × 10^−6^	6.70 × 10^−11^	3.02 × 10^−11^	1.78 × 10^−10^	3.02 × 10^−11^	9.76 × 10^−10^	8.99 × 10^−11^	9.92 × 10^−11^	3.02 × 10^−11^	3.02 × 10^−11^	8.99 × 10^−11^	0.039849	0.304177	\
	Rank	4	12	13	11	10	6	7	5	14	9	8	3	2	1
F10	Avg.	9.83 × 10^3^	4.13 × 10^4^	2.22 × 10^4^	1.47 × 10^4^	2.05 × 10^4^	1.09 × 10^4^	1.51 × 10^4^	1.74 × 10^4^	1.07 × 10^5^	2.22 × 10^4^	2.75 × 10^4^	9.39 × 10^3^	9.27 × 10^3^	**6.81 × 10^3^**
	Std.	4.38 × 10^3^	2.06 × 10^4^	7.42 × 10^3^	7.84 × 10^3^	8.95 × 10^3^	5.11 × 10^3^	6.09 × 10^3^	6.52 × 10^3^	4.42 × 10^4^	1.32 × 10^4^	1.52 × 10^4^	5.02 × 10^3^	4.28 × 10^3^	3.05 × 10^3^
	*p*-value	9.88 × 10^−3^	3.02 × 10^−11^	3.34 × 10^−11^	9.51 × 10^−6^	2.92 × 10^−9^	8.12 × 10^−4^	1.73 × 10^−7^	6.72 × 10^−10^	3.02 × 10^−11^	1.09 × 10^−10^	3.34 × 10^−11^	0.045545	0.016955	\
	Rank	4	13	10	6	9	5	7	8	14	11	12	3	2	1
F11	Avg.	2.43 × 10^3^	2.57 × 10^3^	2.57 × 10^3^	2.55 × 10^3^	2.48 × 10^3^	2.43 × 10^3^	2.49 × 10^3^	2.49 × 10^3^	2.53 × 10^3^	2.51 × 10^3^	2.46 × 10^3^	2.38 × 10^3^	2.38 × 10^3^	**2.37 × 10^3^**
	Std.	39.8	48.8	19.6	39.9	32.4	32	48.2	15.9	14.8	41.2	39.7	21.5	22.6	13.7
	*p*-value	3.16 × 10^−10^	3.02 × 10^−11^	3.02 × 10^−11^	3.02 × 10^−11^	3.02 × 10^−11^	6.70 × 10^−11^	3.02 × 10^−11^	3.02 × 10^−11^	3.02 × 10^−11^	3.02 × 10^−11^	3.02 × 10^−11^	1.95 × 10^−3^	2.32 × 10^−2^	\
	Rank	5	14	13	12	7	4	9	8	11	10	6	3	2	1
F12	Avg.	3104.136	3070.642	3022.532	3093.835	2816.954	2756.675	2942.635	2898.603	2874.587	2902.474	2837.210	2736.769	2737.273	**2717.570**
	Std.	109	116	39.6	90.4	45.4	33.8	84	31	16.3	70.6	40.6	25.8	17.9	22.9
	*p*-value	3.02 × 10^−11^	3.02 × 10^−11^	3.02 × 10^−11^	3.02 × 10^−11^	8.15 × 10^−11^	5.46 × 10^−6^	3.02 × 10^−11^	3.02 × 10^−11^	3.02 × 10^−11^	9.92 × 10^−11^	4.08 × 10^−11^	4.86 × 10^−3^	1.68 × 10^−4^	\
	Rank	14	12	11	13	5	4	10	8	7	9	6	2	3	1
F13	Avg.	**2882.549**	2999.396	3337.453	2919.762	3132.920	2907.474	2899.530	2952.157	2894.800	3029.220	3103.787	2898.501	2897.398	2896.925
	Std.	7.63	41.8	225	19.9	84.1	20.6	18.3	13.9	3.99	53.1	109	16.6	14.5	16.4
	*p*-value	7.60 × 10^−7^	4.98 × 10^−11^	3.02 × 10^−11^	4.64 × 10^−5^	3.02 × 10^−11^	0.228	0.446	3.47 × 10^−10^	0.277	8.15 × 10^−11^	3.02 × 10^−11^	0.0251	0.589	\
	Rank	1	10	14	8	13	7	6	9	2	11	12	5	4	3
F14	Avg.	3247.986	3379.984	3429.615	3366.491	3272.398	3228.606	3272.358	3299.833	**3200.007**	3334.765	3303.191	3238.720	3237.892	3231.053
	Std.	153	94.9	42.5	113	30.5	13.4	28.1	60.4	5.53 × 10^−5^	60.1	42.6	15.3	13.2	15.5
	*p*-value	1.11 × 10^−6^	4.08 × 10^−11^	3.02 × 10^−11^	2.15 × 10^−10^	1.87 × 10^−7^	0.695	2.83 × 10^−8^	2.60 × 10^−8^	3.02 × 10^−11^	8.99 × 10^−11^	2.87 × 10^−10^	0.017649	0.028128	\
	Rank	6	13	14	12	8	2	7	9	1	11	10	5	4	3
F15	Avg.	3810.991	5000.920	4849.046	4472.339	4326.797	3998.324	4161.278	4112.896	4701.157	4442.954	4022.970	3623.276	3615.712	**3507.604**
	Std.	236	489	270	329	249	219	280	166	211	343	169	171	149	149
	*p*-value	2.15 × 10^−6^	3.02 × 10^−11^	3.02 × 10^−11^	3.02 × 10^−11^	3.02 × 10^−11^	6.12 × 10^−10^	1.46 × 10^−10^	4.98 × 10^−11^	3.02 × 10^−11^	3.02 × 10^−11^	1.21 × 10^−10^	1.60 × 10^−3^	5.08 × 10^−3^	\
	Rank	4	14	13	11	9	5	8	7	12	10	6	3	2	1
F16	Avg.	22.1482	1157.662	130.1231	1	7.2981	13.0734	**1**	**1**	228.2632	1	1.3684	**1**	**1**	**1**
	Std.	31.7	1.22 × 10^3^	306	0	16	30.5	0	0	135	5.69 × 10^−13^	2.02	0	0	0
	*p*-value	1.31 × 10^−7^	1.21 × 10^−12^	1.21 × 10^−12^	NaN	1.21 × 10^−12^	5.77 × 10^−11^	NaN	NaN	1.21 × 10^−12^	2.79 × 10^−3^	1.21 × 10^−12^	NaN	NaN	\
	Rank	11	14	12	1	9	10	1	1	13	7	8	1	1	1
F17	Avg.	4.60865	48.24428	13.50236	4.96474	4.78826	5.46781	4.75392	**3.22537**	38.49228	4.34519	4.73527	4.14613	4.13146	4.04666
	Std.	0.532	14.2	5.91	0.113	0.650	1.42	0.333	0.0285	6.70	0.224	0.532	0.177	0.173	0.253
	*p*-value	1.19 × 10^−6^	3.02 × 10^−11^	3.02 × 10^−11^	5.22 × 10^−12^	8.15 × 10^−11^	8.99 × 10^−11^	1.80 × 10^−10^	3.02 × 10^−11^	3.02 × 10^−11^	3.25 × 10^−7^	8.89 × 10^−10^	0.019515	0.018817	\
	Rank	6	14	12	10	9	11	8	1	13	5	7	4	3	2
F18	Avg.	12.31206	7.68058	12.70645	3.40382	12.15974	12.67873	2.05909	10.69658	13.08264	3.47342	8.00827	1.409135	1.590347	**1.381860**
	Std.	1.07	2.92	0.0342	1.41	0.562	0.183	1.35	0.942	0.295	1.94	3.56	2.29 × 10^−7^	0.730	0.104
	*p*-value	3.02 × 10^−11^	3.02 × 10^−11^	3.02 × 10^−11^	3.02 × 10^−11^	3.02 × 10^−11^	3.02 × 10^−11^	2.60 × 10^−8^	3.02 × 10^−11^	3.02 × 10^−11^	5.57 × 10^−10^	3.02 × 10^−11^	0.695	2.23 × 10^−9^	\
	Rank	11	7	13	5	10	12	4	9	14	6	8	2	3	1
F19	Avg.	26.83571	46.74180	38.23376	43.14229	22.06869	23.75134	32.23018	23.40832	22.61743	32.22319	22.46802	13.47014	11.24807	**8.79384**
	Std.	8.60	16.4	6.33	11	8.53	13.9	10	3.31	3.95	12.4	7.84	6.97	4.78	4.55
	*p*-value	2.83 × 10^−10^	4.44 × 10^−11^	2.98 × 10^−11^	2.98 × 10^−11^	2.58 × 10^−8^	5.95 × 10^−8^	4.44 × 10^−11^	1.59 × 10^−10^	3.12 × 10^−10^	1.08 × 10^−10^	3.78 × 10^−9^	2.54 × 10^−4^	0.0118	\
	Rank	9	14	12	13	4	8	11	7	6	10	5	3	2	1
F20	Avg.	1.129713	1.899539	6.586950	1.859639	3.142855	1.231672	1.414871	1.907873	1.172815	1.714809	3.621011	**1.037419**	1.053001	1.055549
	Std.	0.0602	0.516	1.41	0.269	1.16	0.138	0.245	0.104	0.106	0.617	6.08	0.0216	0.0398	0.0319
	*p*-value	3.25 × 10^−7^	3.02 × 10^−11^	3.02 × 10^−11^	3.02 × 10^−11^	3.02 × 10^−11^	3.47 × 10^−10^	2.61 × 10^−10^	3.02 × 10^−11^	1.53 × 10^−5^	3.02 × 10^−11^	3.02 × 10^−11^	9.06 × 10^−3^	0.438	\
	Rank	4	10	14	9	12	6	7	11	5	8	13	1	2	3
F21	Avg.	3.333904	7.560357	6.422529	7.073289	7.177894	3.179001	6.568334	3.895640	7.070256	4.992393	3.695282	1.163341	**1.012581**	1.031128
	Std.	1.33	1.71	1.06	1.59	1.54	1.52	1.94	0.586	1.40	1.54	1.40	0.449	0.0345	0.0479
	*p*-value	6.06 × 10^−11^	2.72 × 10^−11^	2.72 × 10^−11^	2.72 × 10^−11^	2.72 × 10^−11^	2.61 × 10^−10^	2.72 × 10^−11^	2.72 × 10^−11^	2.72 × 10^−11^	2.72 × 10^−11^	2.72 × 10^−11^	0.047485	0.617057	\
	Rank	5	14	9	12	13	4	10	7	11	8	6	3	1	2
F22	Avg.	977.3613	1196.520	1316.158	1024.136	874.9208	756.3315	947.4577	740.3396	1429.732	1032.339	956.2095	460.4976	363.6702	**265.2986**
	Std.	302	346	169	269	287	283	242	145	138	255	279	177	147	165
	*p*-value	9.92 × 10^−11^	4.98 × 10^−11^	3.02 × 10^−11^	4.98 × 10^−11^	1.69 × 10^−9^	5.46 × 10^−9^	8.15 × 10^−11^	2.15 × 10^−10^	3.02 × 10^−11^	3.69 × 10^−11^	9.92 × 10^−11^	0.000163	0.024615	\
	Rank	9	12	13	10	6	5	7	4	14	11	8	3	2	1
F23	Avg.	4.131372	4.408009	4.272127	4.515372	4.216944	3.741861	4.083436	3.830829	4.469262	3.993825	3.800910	2.835498	2.832477	**2.720197**
	Std.	0.440	0.396	0.190	0.478	0.325	0.430	0.401	0.248	0.171	0.391	0.467	0.505	0.470	0.405
	*p*-value	3.34 × 10^−11^	3.02 × 10^−11^	3.02 × 10^−11^	1.96 × 10^−10^	3.02 × 10^−11^	4.20 × 10^−10^	1.09 × 10^−10^	4.50 × 10^−11^	3.02 × 10^−11^	8.99 × 10^−11^	1.96 × 10^−10^	0.559	0.387	\
	Rank	9	12	11	14	10	4	8	6	13	7	5	3	2	1
F24	Avg.	1.236736	1.431654	1.429229	1.411646	1.281101	1.282754	1.337476	1.290872	1.160241	1.289646	1.201894	**1.073908**	1.078166	1.074186
	Std.	0.0862	0.221	0.0889	0.198	0.0669	0.112	0.158	0.0406	0.0274	0.124	0.0751	0.0341	0.0367	0.0321
	*p*-value	7.12 × 10^−9^	7.39 × 10^−11^	3.02 × 10^−11^	6.70 × 10^−11^	4.98 × 10^−11^	2.87 × 10^−10^	6.70 × 10^−11^	3.02 × 10^−11^	7.09 × 10^−8^	1.33 × 10^−10^	3.65 × 10^−8^	0.00152	0.040900	\
	Rank	6	14	13	12	7	8	11	10	4	9	5	1	3	2
F25	Avg.	19.66580	21.10493	21.21701	21.01621	21.34791	21.02717	21.03486	19.48758	21.30001	20.25195	20.74204	14.99284	14.66303	**13.65820**
	Std.	5.07	0.102	0.791	0.0254	0.0797	0.0573	0.0681	4.31	0.07182	3.22	1.84	9.32	9.14	9.80
	*p*-value	0.6	3.02 × 10^−11^	3.02 × 10^−11^	5.97 × 10^−5^	3.02 × 10^−11^	9.52 × 10^−4^	6.77 × 10^−5^	4.74 × 10^−6^	3.02 × 10^−11^	3.65 × 10^−8^	3.02 × 10^−11^	0.0421	0.0309	\
	Rank	5	11	12	8	14	9	10	4	13	6	7	3	2	1
+/=/−		2/2/21	0/0/25	0/0/25	0/1/24	0/0/25	1/2/22	0/3/22	1/1/23	1/1/23	0/0/25	0/0/25	2/4/19	2/5/18	\
Avg. Rank		5.6	12.32	12.52	9.24	9.36	6.12	7.32	7	10.48	9.24	8.28	3.00	2.52	1.40
Final Rank		4	13	14	9	11	5	7	6	12	9	8	3	2	1

**Table 4:** The Friedman test results of HAHA and other algorithms for 25 benchmark functions.

Algorithms	Fun														
	F1	F2	F3	F4	F5	F6	F7	F8	F9	F10	F11	F12	F13		F14
PSO	2.93	3.03	6.77	5.70	3.50	3.63	4.73	4.17	4.13	2.67	5.43	12.77	2.17		4.13
WOA	9.00	11.17	10.67	12.10	11.13	11.47	13.00	12.17	10.43	11.43	12.50	12.23	10.47		11.73
SCA	13.93	13.87	11.83	13.67	13.77	11.77	11.83	9.73	12.43	12.53	12.90	11.73	13.73		13.13
HHO	7.97	8.23	8.53	10.07	7.60	5.67	9.20	6.40	9.70	8.80	11.57	12.47	6.40		11.53
SOA	12.73	8.63	9.80	8.20	8.80	11.20	10.17	8.90	9.57	9.30	7.63	5.37	12.57		7.93
SSA	4.10	4.00	7.10	4.17	6.07	6.63	6.77	4.90	6.37	5.90	4.90	3.50	5.77		4.83
AVOA	3.57	6.83	8.07	8.67	7.23	5.07	6.43	6.80	7.67	7.17	8.53	9.43	5.50		7.60
CryStAl	10.10	6.67	8.80	7.93	9.53	9.17	5.20	8.00	5.67	5.07	8.27	8.37	8.93		8.63
DMOA	7.03	12.10	13.83	10.50	12.23	13.57	10.97	13.87	13.33	13.13	10.87	7.60	4.50		1.47
SCSO	10.97	9.90	7.53	10.07	8.93	8.77	8.97	9.07	9.47	8.73	9.27	8.27	10.80		11.23
GJO	12.27	8.30	5.73	6.80	7.70	10.83	10.50	10.33	9.20	10.27	6.63	6.37	12.20		8.37
AHA	4.23	5.10	2.40	2.83	3.63	2.27	2.47	4.03	2.50	3.23	2.53	2.60	4.40		5.50
AOAHA	3.47	4.37	2.40	2.70	3.17	2.63	2.50	3.83	2.10	3.77	2.33	2.77	3.77		5.00
HAHA	2.70	2.80	1.53	1.60	1.70	2.33	2.27	2.80	2.17	3.00	1.63	1.53	3.80		4.07
Algorithms	Fun											Avg. Rank		Overall Rank	
	F15	F16	F17	F18	F19	F20	F21	F22	F23	F24	F25				
PSO	4.23	8.78	7.00	10.27	8.30	4.47	5.57	8.40	9.17	7.47	4.17	5.74		4	
WOA	12.63	13.80	13.67	7.63	12.20	10.00	11.60	10.43	10.97	11.13	9.03	11.30		13	
SCA	12.77	11.57	11.83	12.97	11.73	13.90	10.30	12.10	9.97	12.40	12.40	12.35		14	
HHO	10.10	4.08	9.03	5.60	12.13	10.33	11.30	9.13	11.70	10.93	6.50	9.00		10	
SOA	9.27	9.70	7.97	10.80	6.50	12.07	11.70	7.20	9.67	8.73	12.50	9.48		11	
SSA	6.10	9.52	9.60	11.30	6.83	5.77	5.60	6.17	6.13	8.77	6.23	6.28		5	
AVOA	7.60	4.08	8.00	3.77	9.47	7.27	10.43	8.17	8.60	9.63	6.20	7.27		6	
CryStAl	7.07	4.08	1.00	8.63	6.83	11.00	6.53	5.83	6.23	9.33	9.07	7.44		7	
DMOA	11.63	12.87	13.30	14.00	7.03	5.00	11.40	13.07	11.47	4.87	11.87	10.46		12	
SCSO	10.03	5.23	5.57	5.37	9.50	8.77	8.17	8.83	7.83	8.63	7.07	8.68		9	
GJO	6.37	9.03	7.87	7.67	6.57	9.67	6.30	8.33	6.37	6.40	11.27	8.45		8	
AHA	2.73	4.08	3.37	1.70	3.55	1.87	2.37	2.57	3.60	2.23	2.60	3.14		3	
AOAHA	2.67	4.08	3.57	3.63	2.53	2.55	1.67	2.11	2.43	1.93	2.53	2.98		2	
HAHA	1.80	4.08	3.23	1.67	1.97	2.35	2.07	1.73	1.70	2.43	2.93	2.40		1	

**Table 5:** The computational time of HAHA and other algorithms for 25 benchmark functions.

Fun	Algorithms													
	PSO	WOA	SCA	HHO	SOA	SSA	AVOA	CryStAl	DMOA	SCSO	GJO	AHA	AOAHA	HAHA
F1	0.78	0.97	1.09	2.69	1.11	1.62	1.59	8.02	7.47	14.94	1.62	1.77	2.85	2.86
F2	0.61	0.73	0.99	2.18	1.23	1.72	1.62	6.72	6.66	14.14	1.68	1.54	2.79	2.96
F3	0.68	0.80	1.00	2.55	1.03	1.44	1.36	7.11	6.75	13.06	1.44	1.68	2.60	2.38
F4	0.89	1.00	1.20	2.89	1.20	1.71	1.64	8.19	8.18	23.90	1.66	1.81	3.13	3.13
F5	0.87	0.94	1.20	3.07	1.22	1.70	1.64	7.85	7.25	19.91	1.64	1.90	3.11	2.84
F6	0.81	0.88	1.16	2.83	1.42	1.91	1.83	8.88	7.86	14.26	1.87	2.07	3.22	3.32
F7	1.06	1.15	1.40	3.39	1.42	1.98	1.87	9.09	7.80	18.78	1.81	1.94	3.34	3.33
F8	0.63	0.73	0.96	2.32	1.01	1.48	1.46	7.47	6.97	13.08	1.56	1.49	2.52	2.65
F9	0.88	1.02	1.25	3.21	1.23	1.75	1.71	8.41	7.81	15.10	1.78	2.16	3.30	3.06
F10	0.76	0.88	1.05	2.40	1.06	1.47	1.53	7.13	6.83	12.63	1.47	1.77	2.60	2.55
F11	1.77	1.94	2.11	5.10	2.13	2.66	2.60	12.09	10.09	23.46	2.75	2.74	5.15	5.05
F12	1.97	2.13	2.36	6.26	2.85	3.31	3.33	14.09	11.74	35.84	2.85	2.93	5.15	5.44
F13	1.86	2.03	2.26	5.38	2.23	2.79	3.18	13.56	11.46	29.12	2.77	3.00	5.33	5.12
F14	2.81	2.96	3.31	7.28	3.06	3.47	3.47	15.94	11.01	16.47	3.74	3.75	6.99	7.07
F15	1.87	1.98	2.16	5.35	2.21	2.69	2.68	12.26	10.18	24.60	2.74	2.75	5.05	5.01
F16	1.09	0.98	1.32	3.59	1.31	1.68	1.54	8.37	10.58	5.11	1.96	2.03	3.39	2.79
F17	0.58	0.59	0.72	2.22	0.99	1.15	1.26	6.50	6.86	7.44	1.16	1.35	2.29	2.10
F18	0.49	0.57	0.71	1.96	0.73	1.17	1.26	6.54	6.82	8.36	1.15	1.52	2.17	2.17
F19	0.48	0.59	0.64	1.86	0.67	1.02	1.20	6.28	7.09	4.99	1.16	1.32	2.19	2.03
F20	0.47	0.58	0.67	2.01	0.67	1.00	1.16	6.18	6.99	4.78	1.05	1.31	2.26	2.09
F21	4.98	5.27	5.33	12.16	4.78	5.13	5.28	22.54	14.82	9.70	5.48	5.51	10.87	11.01
F22	0.48	0.62	0.69	1.94	0.69	1.08	1.16	6.24	6.35	4.52	1.16	1.21	2.17	1.97
F23	0.47	0.58	0.63	1.92	0.66	1.04	1.15	6.21	6.64	4.73	1.17	1.36	2.24	2.12
F24	0.48	0.60	0.64	1.93	0.83	1.27	1.35	6.79	7.36	5.03	1.13	1.20	2.13	1.96
F25	0.49	0.61	0.68	2.08	0.71	1.09	1.26	6.76	7.08	4.94	1.13	1.29	2.33	2.12

**Table 6:** The comparison results of HAHA and other algorithms for CEC 2022 test functions.

F	Index	Algorithms													
		PSO	WOA	SCA	HHO	SOA	SSA	SAO	POA	KOA	SCSO	GJO	AHA	AOAHA	HAHA
F1	Avg.	300	8.83 × 10^3^	786	301	1.06 × 10^3^	300	7.70 × 10^3^	354	1.96 × 10	971	1.67 × 10^3^	300	300	300
	Std.	3.17 × 10^−14^	5.18 × 10^3^	257	0.328	1.69 × 10^3^	3.46 × 10^−10^	2.07 × 10^3^	40.8	6.36 × 10^3^	1.27 × 10^3^	2.04 × 10^3^	6.82 × 10^−10^	1.63 × 10^−11^	5.32 × 10^−11^
	*p*-value	1.08 × 10^−6^	2.76 × 10^−11^	2.76 × 10^−11^	2.76 × 10^−11^	2.76 × 10^−11^	2.76 × 10^−11^	2.76 × 10^−11^	2.76 × 10^−11^	2.76 × 10^−11^	2.76 × 10^−11^	2.76 × 10^−11^	4.67 × 10^−3^	1.42 × 10^−3^	\
	Rank	1	13	8	6	10	5	12	7	14	9	11	1	1	1
F2	Avg.	401	424	454	420	430	410	1.34 × 10^3^	412	646	435	427	400.90136	400.47212	400.87782
	Std.	2.47	31.3	20.5	25.3	55.8	17.6	529	22.4	92.9	33.3	23.9	2.42	1.79	1.67
	*p*-value	0.0184	7.77 × 10^−9^	3.02 × 10^−11^	2.23 × 10^−9^	9.92 × 10^−11^	2.38 × 10^−7^	3.02 × 10^−11^	1.34 × 10^−5^	3.02 × 10^−11^	1.41 × 10^−9^	3.02 × 10^−11^	7.01 × 10^−4^	1.55 × 10^−3^	\
	Rank	4	8	12	7	10	5	14	6	13	11	9	3	1	2
F3	Avg.	606	629	616	627	609	606	646	612	646	610	605	600.00007	600.00004	600.00000
	Std.	5.70	10.7	3.01	11.4	4.89	6.46	8.07	6.40	7.53	6.67	3.62	1.93 × 10^−4^	9.76 × 10^−5^	9.46 × 10^−6^
	*p*-value	2.70 × 10^−11^	2.70 × 10^−11^	2.70 × 10^−11^	2.70 × 10^−11^	2.70 × 10^−11^	2.70 × 10^−11^	2.70 × 10^−11^	2.70 × 10^−11^	2.70 × 10^−11^	2.70 × 10^−11^	2.70 × 10^−11^	2.72 × 10^−3^	8.63 × 10^−3^	\
	Rank	6	12	10	11	7	5	13	9	14	8	4	3	2	1
F4	Avg.	817	836	835	823	820	820	859	815	874	825	824	820	818	815
	Std.	6.86	16.9	5.88	6.75	6.04	8.05	9.26	3.42	10.6	7.19	9.27	7.24	7.31	4.59
	*p*-value	0.145	1.24 × 10^−7^	2.98 × 10^−11^	7.55 × 10^−7^	4.44 × 10^−4^	5.56 × 10^−3^	2.98 × 10^−11^	0.297	2.98 × 10^−11^	2.18 × 10^−7^	5.94 × 10^−5^	1.13 × 10^−3^	0.0160	\
	Rank	3	12	11	8	6	5	13	2	14	10	9	7	4	1
F5	Avg.	908	1.38 × 10^3^	970	1.36 × 10^3^	982	901	1.60 × 10^3^	939	1.92 × 10^3^	1.00 × 10^3^	942	900.09959	900.10497	900.08438
	Std.	33.3	319	26.8	129	50.1	5.15	222	43.3	322	99.1	44.3	0.202	0.387	0.829
	*p*-value	2.55 × 10^−3^	3.00 × 10^−11^	3.00 × 10^−11^	3.00 × 10^−11^	3.67 × 10^−11^	0.0877	3.00 × 10^−11^	2.36 × 10^−10^	3.00 × 10^−11^	3.67 × 10^−11^	6.09 × 10^−10^	0.492	0.0310	\
	Rank	5	12	8	11	9	4	13	6	14	10	7	2	3	1
F6	Avg.	2.96 × 10^3^	3.60 × 10^3^	1.13 × 10^6^	2.73 × 10^3^	1.16 × 10^4^	3.81 × 10^3^	1.11 × 10^7^	1.95 × 10^3^	3.86 × 10^7^	5.08 × 10^3^	6.96 × 10^3^	1804.7947	1804.1257	1803.4043
	Std.	1.63 × 10^3^	1.63 × 10^3^	9.62 × 10^5^	1.11 × 10^3^	5.07 × 10^3^	1.80 × 10^3^	2.37 × 10^7^	89.2	2.74 × 10^7^	2.21 × 10^3^	1.93 × 10^3^	6.36	4.52	4.45
	*p*-value	5.57 × 10^−10^	3.02 × 10^−11^	3.02 × 10^−11^	3.34 × 10^−11^	3.02 × 10^−11^	3.02 × 10^−11^	3.02 × 10^−11^	4.50 × 10^−11^	3.02 × 10^−11^	3.02 × 10^−11^	3.02 × 10^−11^	0.0124	1.15 × 10^−3^	\
	Rank	6	7	12	5	11	8	13	4	14	9	10	3	2	1
F7	Avg.	2.03 × 10^3^	2.06 × 10^3^	2.05 × 10^3^	2.05 × 10^3^	2.04 × 10^3^	2.03 × 10^3^	2.08 × 10^3^	2.03 × 10^3^	2.11 × 10^3^	2.04 × 10^3^	2.03 × 10^3^	2004.2951	2006.3915	2003.6587
	Std.	12.9	20.4	6.94	21.8	12.7	12.6	17.4	8.74	16.9	11.3	11.2	7.99	8.60	7.46
	*p*-value	3.46 × 10^−10^	3.01 × 10^−11^	3.01 × 10^−11^	3.01 × 10^−11^	4.96 × 10^−11^	8.12 × 10^−11^	3.01 × 10^−11^	1.46 × 10^−10^	3.01 × 10^−11^	3.01 × 10^−11^	4.96 × 10^−11^	1.39 × 10^−3^	0.0122	\
	Rank	5	12	11	10	8	6	13	4	14	9	7	2	3	1
F8	Avg.	2.22 × 10^3^	2.23 × 10^3^	2.23 × 10^3^	2.23 × 10^3^	2.23 × 10^3^	2.23 × 10^3^	2.24 × 10^3^	2.22 × 10^3^	2.26 × 10^3^	2.22 × 10^3^	2.23 × 10^3^	2214.3083	2214.7677	2209.7614
	Std.	5.05	6.39	3.42	5.77	2.05	4.73	13.1	8.75	16	4.22	4.19	9.11	8.67	9.44
	*p*-value	3.09 × 10^−6^	1.09 × 10^−10^	1.09 × 10^−10^	3.02 × 10^−11^	3.02 × 10^−11^	1.46 × 10^−10^	3.02 × 10^−11^	1.89 × 10^−4^	3.02 × 10^−11^	1.33 × 10^−10^	4.20 × 10^−10^	0.0297	1.05 × 10^−3^	\
	Rank	5	12	11	10	9	7	13	4	14	6	8	2	3	1
F9	Avg.	2.49 × 10^3^	2.54 × 10^3^	2.55 × 10^3^	2.55 × 10^3^	2.56 × 10^3^	2.53 × 10^3^	2.73 × 10^3^	2.53 × 10^3^	2.69 × 10^3^	2.57 × 10^3^	2.57 × 10^3^	2529.2844	2529.2843	2529.2842
	Std.	34.8	16.2	13.2	43.4	41.8	26.8	27.5	0.486	37.6	41.6	37.6	9.43 × 10^−12^	1.26 × 10^−11^	8.24 × 10^−12^
	*p*-value	1.43 × 10^−10^	3.01 × 10^−11^	3.01 × 10^−11^	3.01 × 10^−11^	3.01 × 10^−11^	3.01 × 10^−11^	3.01 × 10^−11^	3.01 × 10^−11^	3.01 × 10^−11^	3.01 × 10^−11^	3.01 × 10^−11^	0.0273	1.93 × 10^−3^	\
	Rank	1	7	9	8	10	6	14	5	13	11	12	4	3	2
F10	Avg.	2.55 × 10^3^	2.54 × 10^3^	2.50 × 10^3^	2.55 × 10^3^	2.50 × 10^3^	2.50 × 10^3^	2.74 × 10^3^	2.50 × 10^3^	2.55 × 10^3^	2.52 × 10^3^	2.52 × 10^3^	2500.3118	2500.3111	2500.3007
	Std.	61.8	63.3	0.382	66.8	22.2	21.5	203	0.233	50.5	46.9	45	0.0777	0.0718	0.0641
	*p*-value	1.44 × 10^−3^	4.98 × 10^−11^	3.02 × 10^−11^	3.69 × 10^−11^	1.96 × 10^−10^	3.57 × 10^−6^	3.02 × 10^−11^	2.57 × 10^−7^	3.02 × 10^−11^	1.43 × 10^−5^	8.84 × 10^−7^	0.340	0.483	\
	Rank	11	10	5	12	7	6	14	4	13	9	8	3	2	1
F11	Avg.	2.62 × 10^3^	2.78 × 10^3^	2.77 × 10^3^	2.77 × 10^3^	2.75 × 10^3^	2.63 × 10^3^	3.26 × 10^3^	2.67 × 10^3^	2.99 × 10^3^	2.80 × 10^3^	2.78 × 10^3^	2600	2600	2600
	Std.	77.1	179	10.2	186	82.4	82.7	297	100	120	198	136	3.87 × 10^−13^	4.85 × 10^−13^	3.68 × 10^−13^
	*p*-value	0.255	4.10 × 10^−12^	4.10 × 10^−12^	4.10 × 10^−12^	4.10 × 10^−12^	4.10 × 10^−12^	4.10 × 10^−12^	4.10 × 10^−12^	4.10 × 10^−12^	4.10 × 10^−12^	4.10 × 10^−12^	0.721	0.592	\
	Rank	4	11	8	9	7	5	14	6	13	12	10	1	1	1
F12	Avg.	2.89 × 10^3^	2.89 × 10^3^	2.87 × 10^3^	2.88 × 10^3^	2863.764	2862.407	3.02 × 10^3^	2.86 × 10^3^	2.88 × 10^3^	2.87 × 10^3^	2.87 × 10^3^	2865.5126	2865.4835	2863.7415
	Std.	35.5	30.2	1.49	25	1.42	1.99	72.61	2.59	10.3	2.11	6.53	1.64	1.44	0.996
	*p*-value	0.663	8.32 × 10^−8^	3.48 × 10^−9^	3.18 × 10^−9^	7.64 × 10^−5^	2.59 × 10^−8^	3.00 × 10^−11^	0.0271	3.00 × 10^−11^	0.559	0.133	0.0209	0.0149	\
	Rank	12	13	9	11	3	1	14	4	10	5	8	7	6	2
+/=/−		1/1/10	0/0/12	0/0/12	0/0/12	0/0/12	1/0/11	0/0/12	0/0/12	0/0/12	0/0/12	0/0/25	0/2/10	1/2/9	\
Avg. Rank		5.250	10.750	9.500	9.000	8.083	5.250	13.333	5.083	13.333	9.083	8.583	3.167	2.583	1.250
Final Rank		5	12	11	9	7	5	13	4	13	10	8	3	2	1

## Data Availability

All data generated or analyzed during the study are included in this published article.

## Appendix A. Twenty-Five Benchmark Functions

**Table A1:** The 25 benchmark functions.

Function Type	Function Name	Dim	Search Range	Optimal Value
Uni-modal functions	F1: Shifted and Rotated Bent Cigar Function (CEC 2017 F1)	30	[−100,100]	100
Multi-modal functions	F2: Shifted and Rotated Rastrigin’s Function (CEC 2014 F9)	30	[−100,100]	900
	F3: Shifted and Rotated Expanded Scaffer’s F6 Function (CEC 2014 F16)	30	[−100,100]	1600
	F4: Shifted and Rotated Rastrigin’s Function (CEC 2017 F5)	30	[−100,100]	500
	F5: Shifted and Rotated Non-Continuous Rastrigin’s Function (CEC 2017 F8)	30	[−100,100]	800
Hybrid functions	F6: Hybrid Function 1 (N = 3) (CEC 2017 F11)	30	[−100,100]	1100
	F7: Hybrid Function 2 (N = 4) (CEC 2017 F14)	30	[−100,100]	1400
	F8: Hybrid Function 3 (N = 4) (CEC 2014 F20)	30	[−100,100]	2000
	F9: Hybrid Function 4 (N = 5) (CEC 2017 F18)	30	[−100,100]	1800
	F10: Hybrid Function 5 (N = 5) (CEC 2014 F21)	30	[−100,100]	2100
Composition functions	F11: Composition Function 1 (N = 3) (CEC 2017 F21)	30	[−100,100]	2100
	F12: Composition Function 2 (N = 4) (CEC 2017 F23)	30	[−100,100]	2300
	F13: Composition Function 3 (N = 5) (CEC 2017 F25)	30	[−100,100]	2500
	F14: Composition Function 4 (N = 6) (CEC 2017 F27)	30	[−100,100]	2700
	F15: Composition Function 5 (N = 3) (CEC 2017 F29)	30	[−100,100]	2900
Fixed dimensional functions	F16: Storn’s Chebyshev Polynomial Fitting Problem (CEC 2019 F1)	9	[−8192,8192]	1
	F17: Inverse Hilbert Matrix Problem (CEC 2019 F2)	16	[−16,384,16,384]	1
	F18: Lennard-Jones Minimum Energy Cluster (CEC 2019 F3)	18	[−4,4]	1
	F19: Rastrigin’s Function (CEC 2019 F4)	10	[−100,100]	1
	F20: Griewangk’s Function (CEC 2019 F5)	10	[−100,100]	1
	F21: Weierstrass Function (CEC 2019 F6)	10	[−100,100]	1
	F22: Modified Schwefel’s Function (CEC 2019 F7)	10	[−100,100]	1
	F23: Expanded Schaffer’s F6 Function (CEC 2019 F8)	10	[−100,100]	1
	F24: Happy Cat Function (CEC 2019 F9)	10	[−100,100]	1
	F25: Ackley Function (CEC 2019 F10)	10	[−100,100]	1

## Appendix B. Proof of Theorems in Section 4

### Appendix B.1. Proof of Theorem 1

**Proof.** If the *j*-segment and the *j* + 1-segment curve of CSGC–Ball curves meet the G^1^ continuity condition at the connection point *u_j_*, then G^0^ should be continuous first, that is,

(A1)
$$P_{3,j}=P_{0,j+1}.$$

Furthermore, the two curves should have the same unit tangent vector at node *u_j_*, which is

(A2) $$kP(u_{j}^{-})=P(u_{j}^{+}),k>0,$$

From the endpoint properties of the SGC–Ball curves, it is known that

(A3) $$\left\{\begin{array}{l} P^{\prime }(u_{j}^{-})=\frac{1}{h_{j}}(2-\omega (\lambda_{3,j}-1))(P_{3,j}-P_{2,j}),\\ P(u_{j}^{+})=\frac{1}{h_{j+1}}(2-\omega (\lambda_{1,j+1}-1))(P_{1,j+1}-P_{0,j+1}). \end{array}\right.$$

Substituting the above formula into Equation (A2), we can arrange to obtain

(A4) $$P_{1,j+1}=\frac{kh_{j+1}(2-\omega (\lambda_{3,j}-1))}{h_{j}(2-\omega (\lambda_{1,j+1}-1))}(P_{3,j}-P_{2,j})+P_{0,j+1}.$$

where k>0 is an arbitrary constant. Theorem 1 is proved. □

### Appendix B.2. Proof of Theorem 2

**Proof.** If the *j*-segment and the *j* + 1-segment curves of the CSGC–Ball curves meet the G^2^ continuity condition at node *u_j_*, then the G^1^ continuity should be satisfied first, which is given by Theorem 1.

Let **D**_1_ and **D**_2_ be the subnormal vectors of the *j*-segment and *j* + 1-segment SGC–Ball curves at node *u_j_*, respectively. Then

(A5) $$D_{1}=P^{\prime }(u_{j}^{-})\times P^{\prime \prime }(u_{j}^{-}),\;D_{2}=P^{\prime }(u_{j}^{+})\times P^{\prime \prime }(u_{j}^{+})$$

To satisfy G^2^ continuity, **D**_1_ and **D**_2_ must have the same tangent vector at node *u_j_*. As is known from Equation (A2), $P^{\prime }(u_{j}^{-}),P^{\prime \prime }(u_{j}^{-}),P^{\prime }(u_{j}^{+}),P^{\prime \prime }(u_{j}^{+})$ are collinear, that is, **D**_1_ and **D**_2_ have the same direction at *u_j_*, that is

(A6) $$P(u_{j}^{+})=\alpha P^{\prime \prime }(u_{j}^{-})+\beta P^{\prime }(u_{j}^{-})$$

Among them, α,β are arbitrary constants, and α>0.

Let $k(u_{j}^{-}),k(u_{j}^{+})$ be the curvatures of the *j*-segment and *j* + 1-segment curves at the junction *u_j_*, respectively. According to the curvature formula, we have

(A7) $$k(u_{j}^{-})=\frac{\left|P^{\prime }(u_{j}^{-})\times P^{\prime \prime }(u_{j}^{-})\right|}{|P^{\prime }(u_{j}^{-}){|}^{3}},\;k(u_{j}^{+})=\frac{\left|P^{\prime }(u_{j}^{+})\times P^{\prime \prime }(u_{j}^{+})\right|}{|P^{\prime }(u_{j}^{+}){|}^{3}}$$

if the G^2^ continuity condition is satisfied. Substituting Equations (A2) and (A6) into the above Equation (A7), there are

(A8) $$\begin{array}{c} k(u_{j}^{+})=\frac{\left|P^{\prime }(u_{j}^{+})\times P^{\prime \prime }(u_{j}^{+})\right|}{|P^{\prime }(u_{j}^{+}){|}^{3}}=\frac{\left|kP^{\prime }(u_{j}^{-})\times (\alpha P^{\prime \prime }(u_{j}^{-})+\beta P^{\prime }(u_{j}^{-}))\right|}{|\lambda P^{\prime }(u_{j}^{-}){|}^{3}}\\ \;\;\;\;\;=\frac{\alpha |P^{\prime }(u_{j}^{-})\times P^{\prime \prime }(u_{j}^{-})|+\beta |P^{\prime }(u_{j}^{-})\times P^{\prime }(u_{j}^{-})|}{k^{2}|P^{\prime }(u_{j}^{-}){|}^{3}}=\frac{\alpha |P^{\prime }(u_{j}^{-})\times P^{\prime \prime }(u_{j}^{-})|}{k^{2}|P^{\prime }(u_{j}^{-}){|}^{3}}. \end{array}$$

From Equation (A8), when $\alpha =k^{2}$, then $k(u_{j}^{-})=k(u_{j}^{+})$. Then, Equation (A5) can be written as:

(A9) $$P^{\prime \prime }(u_{j}^{+})=k^{2}P^{\prime \prime }(u_{j}^{-})+\beta P^{\prime }(u_{j}^{-})$$

From the endpoint properties of the SGC–Ball curves, the second derivative of the *j*-segment and *j* + 1-segment SGC–Ball curves at the junction *u_j_* can be written as

(A10) $$\left\{\begin{array}{l} P^{\prime \prime }(u_{j}^{-})=\frac{2}{h_{j}^{2}}P_{0,j}+\frac{2}{h_{j}^{2}}(\omega (\lambda_{2,j}-2)+2)P_{1,j}\\ \;\;\;+\frac{2}{h_{j}^{2}}(\omega (3\lambda_{3,j}-\lambda_{2,j}-1)-4)P_{2,j}-\frac{2}{h_{j}^{2}}(3\omega (\lambda_{3,j}-1)-1)P_{3,j},\\ P^{\prime \prime }(u_{j}^{+})=-\frac{2}{h_{j+1}^{2}}(3\omega (\lambda_{1,j+1}-1)-1)P_{0,j+1}+\frac{2}{h_{j+1}^{2}}(\omega (3\lambda_{1,j+1}+\lambda_{2,j+1}-5)-4)P_{1,j+1}\\ \;\;\;-\frac{2}{h_{j+1}^{2}}(\omega (\lambda_{2,j+1}-2)-2)P_{2,j+1}+\frac{2}{h_{j+1}^{2}}P_{3,j+1}. \end{array}\right.$$

By substituting Equations (A3) and (A10) into Equation (A9), the following formula is obtained:

(A11) $$\begin{array}{l} P_{2,j+1}=\frac{2k^{2}h_{j+1}^{2}(3\omega (\lambda_{3,j}-1)-1)-\beta h_{j}h_{j+1}^{2}(2-\omega (\lambda_{3,j}-1))}{\;2h_{j}^{2}(\omega (\lambda_{2,j+1}-2)-2)}P_{3,j}\\ \;\;\;\;+\frac{-2k^{2}h_{j+1}^{2}(\omega (3\lambda_{3,j}-\lambda_{2,j}-1)-4)+\beta h_{j}h_{j+1}^{2}(2-\omega (\lambda_{3,j}-1))}{\;2h_{j}^{2}(\omega (\lambda_{2,j+1}-2)-2)}P_{2,j}\\ \;\;\;\;-\frac{k^{2}h_{j+1}^{2}(\omega (\lambda_{2,j}-2)+2)}{\;h_{j}^{2}(\omega (\lambda_{2,j+1}-2)-2)}P_{1,j}-\frac{k^{2}h_{j+1}^{2}}{\;h_{j}^{2}(\omega (\lambda_{2,j+1}-2)-2)}P_{0,j}\\ \;\;\;\;-\frac{\left(3\omega (\lambda_{1,j+1}-1)-1\right)}{\;\omega (\lambda_{2,j+1}-2)-2}P_{0,j+1}+\frac{\omega (3\lambda_{1,j+1}+\lambda_{2,j+1}-5)-4}{\;\omega (\lambda_{2,j+1}-2)-2}P_{1,j+1}+\frac{1}{\;\omega (\lambda_{2,j+1}-2)-2}P_{3,j+1}. \end{array}$$

where k>0,β is an arbitrary constant. G^2^ is continuous at *u_j_* if Equations (A1), (A4), and (A11) are satisfied. Then, the SGC–Ball curves of *j*-segment and *j* + 1-segment are G^2^ continuous at *u_j_*. Theorem 2 is proved. □

## Appendix C

**Table A2:** Optimal shape parameters and energy values of the CSGC–Ball curves of the overall G^2^ splicing.

Algorithm	Optimal Shape Parameters									*E*
PSO	ω	0.64887919								66.2849
		*j* = 1	*j* = 2	*j* = 3	*j* = 4	*j* = 5	*j* = 6	*j* = 7	*j* = 8	
	$\lambda_{1.j}$	1.15185	1.37390	1.32772	1.55349	1.11589	1.29393	1.46232	1.54878	
	$\lambda_{2.j}$	2.06512	1.96168	2.05905	1.80837	1.98417	1.64995	2.36528	1.96000	
	$\lambda_{3.j}$	1.32390	1.28802	1.47045	1.45026	1.32576	1.06542	1.46548	1.16265	
WOA	ω	0.61246533								42.3460
		*j* = 1	*j* = 2	*j* = 3	*j* = 4	*j* = 5	*j* = 6	*j* = 7	*j* = 8	
	$\lambda_{1.j}$	1.86538	2.53608	2.80315	2.74539	1.97669	2.50878	2.73656	2.61410	
	$\lambda_{2.j}$	2.82767	2.10635	2.18380	2.26413	2.65508	2.20090	2.70274	2.71869	
	$\lambda_{3.j}$	2.47729	2.63008	2.90391	2.23474	2.48893	2.59616	2.97236	2.24699	
SCA	ω	0.44943302								60.6286
		*j* = 1	*j* = 2	*j* = 3	*j* = 4	*j* = 5	*j* = 6	*j* = 7	*j* = 8	
	$\lambda_{1.j}$	1.53812	1.21378	2.25093	1.89868	1.72244	1.40985	2.50022	2.24194	
	$\lambda_{2.j}$	0.28967	0.22890	0.34591	0.12716	0.19957	0.37379	0.60330	0.37674	
	$\lambda_{3.j}$	2.09452	2.25425	2.32298	2.21089	1.27993	2.21361	2.43706	1.85464	
HHO	ω	0.58330802								42.8444
		*j* = 1	*j* = 2	*j* = 3	*j* = 4	*j* = 5	*j* = 6	*j* = 7	*j* = 8	
	$\lambda_{1.j}$	2.26112	2.66245	2.77293	2.75090	2.27722	2.72139	2.79630	2.64388	
	$\lambda_{2.j}$	2.10014	2.04252	2.10340	2.23652	2.31288	2.04330	2.46973	2.39952	
	$\lambda_{3.j}$	2.59681	2.71575	2.89981	2.36353	2.69098	2.67674	2.94551	2.39238	
GWO	ω	0.59802310								42.9182
		*j* = 1	*j* = 2	*j* = 3	*j* = 4	*j* = 5	*j* = 6	*j* = 7	*j* = 8	
	$\lambda_{1.j}$	1.89364	2.73443	2.87954	2.83924	1.94839	2.80668	2.77928	2.63251	
	$\lambda_{2.j}$	0.78809	0.87901	1.71278	1.69333	0.90586	0.45850	2.82386	0.49710	
	$\lambda_{3.j}$	2.45587	2.61126	2.90566	2.20632	2.42842	2.42752	2.99789	2.28221	
HAHA	ω	0.77204510								41.7970
		*j* = 1	*j* = 2	*j* = 3	*j* = 4	*j* = 5	*j* = 6	*j* = 7	*j* = 8	
	$\lambda_{1.j}$	1.72569	2.47537	2.45510	2.42984	1.76746	2.45557	2.36967	2.30659	
	$\lambda_{2.j}$	1.96822	1.67667	2.11353	1.88233	2.03359	1.31535	2.71346	1.80763	
	$\lambda_{3.j}$	2.23051	2.21689	2.53882	1.94375	2.27044	2.11102	2.64168	1.98074	

## Appendix D

**Table A3:** Optimal shape parameters and energy values of the CSGC–Ball curves of mixed G^0^, G^1^, and G^2^ splicing.

Algorithm	Optimal Shape Parameters									*E*
PSO	ω	8.165946× 10^−17^								379.882
		*j* = 1	*j* = 2	*j* = 3	*j* = 4	*j* = 5	*j* = 6	*j* = 7	*j* = 8	
	$\lambda_{1.j}$	0.01199	0.68281	0.37140	−0.1024	−0.3511	0.35437	−0.3069	−0.0002	
	$\lambda_{2.j}$	1.90894	2.26708	2.15170	1.94534	1.88223	2.14267	1.77331	2.11611	
	$\lambda_{3.j}$	−0.1358	0.12068	−0.1542	0.30099	0.33231	0.35239	0.12931	−0.1755	
		*j* = 9	*j* = 10	*j* =11	*j* = 12	*j* = 13	*j* = 14	*j* = 15	*j* = 16	
	$\lambda_{1.j}$	−0.2375	−0.1540	−0.0842	−0.0511	0.46105	0.25110	0.01922	−0.1765	
	$\lambda_{2.j}$	2.21089	1.87741	1.84059	1.92628	1.63868	2.20693	2.11329	1.87746	
	$\lambda_{3.j}$	0.10939	−0.1249	−0.0502	0.24929	−0.1074	−0.0390	0.08777	−0.1168	
		*j* = 17	*j* = 18	*j* =19	*j* = 20	*j* = 21	*j* = 22	*j* = 23	*j* = 24	
	$\lambda_{1.j}$	0.04473	−0.1061	−0.0101	0.22035	−0.2502	0.15433	0.17569	−0.0559	
	$\lambda_{2.j}$	1.99198	2.12004	2.12426	1.88229	1.90081	1.89130	1.87136	1.81734	
	$\lambda_{3.j}$	−0.0062	0.14957	0.17624	0.03901	0.01131	−0.1524	−0.1873	0.07681	
		*j* = 25	*j* = 26	*j* =27	*j* = 28	*j* = 29				
	$\lambda_{1.j}$	0.22829	−0.2374	0.02877	0.18938	0.08976				
	$\lambda_{2.j}$	1.96420	2.33591	2.08569	1.93850	1.98105				
	$\lambda_{3.j}$	0.04001	−0.1362	−0.0880	−0.1776	0.22704				
WOA	ω	0.56143072								184.656
		*j* = 1	*j* = 2	*j* = 3	*j* = 4	*j* = 5	*j* = 6	*j* = 7	*j* = 8	
	$\lambda_{1.j}$	2.85218	2.87138	2.82369	2.89281	2.98078	2.83488	2.88864	2.86913	
	$\lambda_{2.j}$	2.35992	2.05760	2.36563	1.84918	2.34436	2.21655	2.30250	2.16827	
	$\lambda_{3.j}$	2.89427	2.90698	2.93313	2.81194	2.91938	2.87946	2.87397	2.86274	
		*j* = 9	*j* = 10	*j* =11	*j* = 12	*j* = 13	*j* = 14	*j* = 15	*j* = 16	
	$\lambda_{1.j}$	2.87848	2.87460	2.84069	2.87111	2.86491	2.86395	2.86615	2.87630	
	$\lambda_{2.j}$	2.16549	2.21958	2.33277	2.16387	1.82327	2.08353	2.14077	1.87726	
	$\lambda_{3.j}$	2.85025	2.85099	2.89884	2.81336	2.85745	2.89370	2.89365	2.89385	
		*j* = 17	*j* = 18	*j* =19	*j* = 20	*j* = 21	*j* = 22	*j* = 23	*j* = 24	
	$\lambda_{1.j}$	2.84521	2.85292	2.78854	2.84806	2.85620	2.82256	2.82369	2.81984	
	$\lambda_{2.j}$	2.20087	2.39431	2.24645	2.64762	2.45415	2.27135	2.58449	2.39753	
	$\lambda_{3.j}$	2.87404	2.88912	2.77618	2.85626	2.86230	2.89564	2.87897	2.87074	
		*j* = 25	*j* = 26	*j* =27	*j* = 28	*j* = 29				
	$\lambda_{1.j}$	2.78441	2.86354	2.86487	2.84957	2.85947				
	$\lambda_{2.j}$	2.59021	2.35955	2.00985	2.34287	2.20680				
	$\lambda_{3.j}$	2.84430	2.91575	2.87652	2.92195	2.86763				
SCA	ω	0.01240168								378.441
		*j* = 1	*j* = 2	*j* = 3	*j* = 4	*j* = 5	*j* = 6	*j* = 7	*j* = 8	
	$\lambda_{1.j}$	−1.0570	−0.3385	0.23026	0.08677	0.60017	−0.1256	−0.5899	−0.0320	
	$\lambda_{2.j}$	2.15431	1.46926	1.64490	1.50380	2.03020	1.77393	1.23516	1.80551	
	$\lambda_{3.j}$	0.52234	0.12062	−0.4202	−0.2678	−0.4550	−0.0387	−0.2334	−0.0960	
		*j* = 9	*j* = 10	*j* =11	*j* = 12	*j* = 13	*j* = 14	*j* = 15	*j* = 16	
	$\lambda_{1.j}$	0.45369	0.01261	−0.4439	−0.1557	−0.0764	−0.0760	−0.3289	0.17971	
	$\lambda_{2.j}$	1.50170	1.45567	1.64068	1.91496	1.93461	2.28278	1.55732	1.72778	
	$\lambda_{3.j}$	−0.2907	−0.2423	0.01349	−0.3109	−0.1890	−0.2911	−0.0978	0.77774	
		*j* = 17	*j* = 18	*j* =19	*j* = 20	*j* = 21	*j* = 22	*j* = 23	*j* = 24	
	$\lambda_{1.j}$	0.49273	0.25800	−0.1537	−0.1686	0.12123	−0.4168	0.51629	0.29765	
	$\lambda_{2.j}$	1.49014	2.09171	1.38876	1.95450	1.95912	1.58009	1.86773	1.87182	
	$\lambda_{3.j}$	−0.0568	0.03448	−0.1341	0.47338	−0.8309	−0.2569	0.12387	−0.7595	
		*j* = 25	*j* = 26	*j* =27	*j* = 28	*j* = 29				
	$\lambda_{1.j}$	0.06529	0.53273	0.11868	0.01192	−0.1921				
	$\lambda_{2.j}$	1.53690	2.10757	1.98951	1.84692	2.61408				
	$\lambda_{3.j}$	−0.2140	−0.2751	0.63215	−0.3338	−0.5503				
HHO	ω	0.53141245								184.025
		*j* = 1	*j* = 2	*j* = 3	*j* = 4	*j* = 5	*j* = 6	*j* = 7	*j* = 8	
	$\lambda_{1.j}$	2.98076	2.98442	2.98010	2.97835	2.99602	2.98160	2.98318	2.97598	
	$\lambda_{2.j}$	1.98222	2.00455	2.07102	2.00812	2.03681	1.99617	2.02540	1.98957	
	$\lambda_{3.j}$	2.98414	2.99028	2.98943	2.98353	2.97914	2.98232	2.98244	2.98264	
		*j* = 9	*j* = 10	*j* =11	*j* = 12	*j* = 13	*j* = 14	*j* = 15	*j* = 16	
	$\lambda_{1.j}$	2.98423	2.97553	2.97932	2.98584	2.98094	2.98193	2.98142	2.98279	
	$\lambda_{2.j}$	1.92067	1.96532	2.05272	1.94200	1.98074	2.02657	2.03130	1.92533	
	$\lambda_{3.j}$	2.97530	2.97999	2.98822	2.96541	2.97992	2.98762	2.98329	2.98282	
		*j* = 17	*j* = 18	*j* =19	*j* = 20	*j* = 21	*j* = 22	*j* = 23	*j* = 24	
	$\lambda_{1.j}$	2.97561	2.98808	2.97989	2.98418	2.97869	2.98229	2.97976	2.98119	
	$\lambda_{2.j}$	2.05583	2.06593	2.02464	1.99876	1.95430	1.96503	1.98384	1.95259	
	$\lambda_{3.j}$	2.98265	2.98353	2.98228	2.98389	2.98242	2.98294	2.98386	2.98094	
		*j* = 25	*j* = 26	*j* =27	*j* = 28	*j* = 29				
	$\lambda_{1.j}$	2.98291	2.98321	2.98492	2.98132	2.97698				
	$\lambda_{2.j}$	1.99819	1.99420	2.01433	1.95868	2.06611				
	$\lambda_{3.j}$	2.97534	2.98722	2.97132	2.98569	2.98160				
SMA	ω	0.85577335								208.323
		*j* = 1	*j* = 2	*j* = 3	*j* = 4	*j* = 5	*j* = 6	*j* = 7	*j* = 8	
	$\lambda_{1.j}$	2.13464	2.13464	2.13464	2.13464	2.13464	2.13464	2.13464	2.13464	
	$\lambda_{2.j}$	3.42309	3.42309	3.42309	3.42309	3.42309	3.42309	3.42309	3.42309	
	$\lambda_{3.j}$	2.13464	2.13464	2.13464	2.13464	2.13464	2.13464	2.13464	2.13464	
		*j* = 9	*j* = 10	*j* =11	*j* = 12	*j* = 13	*j* = 14	*j* = 15	*j* = 16	
	$\lambda_{1.j}$	2.13464	2.13464	2.13464	2.13464	2.13464	2.13464	2.13464	2.13464	
	$\lambda_{2.j}$	3.42309	3.42309	3.42309	3.42309	3.42309	3.42309	3.42309	3.42309	
	$\lambda_{3.j}$	2.13464	2.13464	2.13464	2.13464	2.13464	2.13464	2.13464	2.13464	
		*j* = 17	*j* = 18	*j* =19	*j* = 20	*j* = 21	*j* = 22	*j* = 23	*j* = 24	
	$\lambda_{1.j}$	2.13464	2.13464	2.13464	2.13464	2.13464	2.13464	2.13464	2.13464	
	$\lambda_{2.j}$	3.42309	3.42309	3.42309	3.42309	3.42309	3.42309	3.42309	3.42309	
	$\lambda_{3.j}$	2.13464	2.13464	2.13464	2.13464	2.13464	2.13464	2.13464	2.13464	
		*j* = 25	*j* = 26	*j* =27	*j* = 28	*j* = 29				
	$\lambda_{1.j}$	2.13464	2.13464	2.13464	2.13464	2.13464				
	$\lambda_{2.j}$	3.42309	3.42309	3.42309	3.42309	3.42309				
	$\lambda_{3.j}$	2.13464	2.13464	2.13464	2.13464	2.13464				
HAHA	ω	0.75838681								182.437
		*j* = 1	*j* = 2	*j* = 3	*j* = 4	*j* = 5	*j* = 6	*j* = 7	*j* = 8	
	$\lambda_{1.j}$	2.32285	2.39393	2.24157	2.38538	2.74613	2.39668	2.35229	2.37876	
	$\lambda_{2.j}$	2.07657	1.87513	2.00551	1.06112	1.47205	1.90181	2.12946	1.97447	
	$\lambda_{3.j}$	2.44915	2.49225	2.59108	1.58380	1.73668	2.31265	2.42312	2.34776	
		*j* = 9	*j* = 10	*j* =11	*j* = 12	*j* = 13	*j* = 14	*j* = 15	*j* = 16	
	$\lambda_{1.j}$	2.38142	2.33024	2.36332	2.38664	2.41047	2.37189	2.35745	2.41375	
	$\lambda_{2.j}$	1.96577	2.14084	2.09773	1.88414	1.75302	1.95039	2.08764	1.71225	
	$\lambda_{3.j}$	2.31657	2.29552	2.38640	2.25331	2.33960	2.41308	2.41769	2.36731	
		*j* = 17	*j* = 18	*j* =19	*j* = 20	*j* = 21	*j* = 22	*j* = 23	*j* = 24	
	$\lambda_{1.j}$	2.35154	2.21571	2.36552	2.40253	2.31975	2.32859	2.27686	2.39248	
	$\lambda_{2.j}$	2.13212	2.97383	1.95326	1.86995	2.30571	2.34025	2.44898	1.85148	
	$\lambda_{3.j}$	2.36369	2.82544	2.29492	2.40542	2.37088	2.68900	2.42857	2.40000	
		*j* = 25	*j* = 26	*j* =27	*j* = 28	*j* = 29				
	$\lambda_{1.j}$	2.27982	2.28423	2.40584	2.33562	2.29302				
	$\lambda_{2.j}$	2.45719	2.76197	1.53374	2.27599	2.09232				
	$\lambda_{3.j}$	2.34878	2.60824	2.26335	2.67179	2.43301				

## References

[1] Shi F.Z. (2001). Computer Aided Geometric Design and Nonuniform Rational B-Splines: CAGD & NURBS.

[2] Wang G.J., Wang G.Z., Zheng J.M. (2001). Computer Aided Geometric Design.

[3] Hu G., Dou W.T., Wang X.F., Abbas M. (2022). An enhanced chimp optimization algorithm for optimal degree reduction of Said–Ball curves. Math. Comput. Simul..

[4] Consurf A.A. (1974). Part one: Introduction of the conic lofting tile. Comput.-Aided Des..

[5] Wang G.J. (1987). Ball curve of high degree and its geometric properties. Appl. Math. A J. Chin. Univ..

[6] Said H.B. (1989). A generalized ball curve and its recursive algorithm. ACM Trans. Graph. (TOG).

[7] Hu S.M., Wang G.Z., Jin T.G. (1996). Properties of two types of generalized Ball curves. Comput.-Aided Des..

[8] Othlnan W., Goldman R.N. (1997). The dual basis functions for the generalized ball basis of odd degree. Comput. Aided Geom. Des..

[9] Xi M.C. (1997). Dual basis of Ball basis function and its application. Comput. Math..

[10] Ding D.Y., Li M. (2000). Properties and applications of generalized Ball curves. Chin. J. Appl. Math..

[11] Jiang P., Wu H. (2004). Dual basis of Wang-Ball basis function and its application. J. Comput. Aided Des. Graph..

[12] Hu S.M., Jin T.G. Degree reductive approximation of Bézier curves. Proceedings of the Eighth Annual Symposium on Computational Geometry.

[13] Wu H.Y. (2000). Two new kinds of generalized Ball curves. J. Appl. Math..

[14] Wang C.W. (2008). Extension of cubic Ball curve. J. Eng. Graph..

[15] Wang C.W. (2009). The extension of the quartic Wang-Ball curve. J. Eng. Graph..

[16] Yan L.L., Zhang W., Wen R.S. (2011). Two types of shape-adjustable fifth-order generalized Ball curves. J. Eng. Graph..

[17] Hu G.S., Wang D., Yu A.M. (2009). Construction and application of 2m+2 degree Ball curve with shape parameters. J. Eng. Graph..

[18] Xiong J., Guo Q.W. (2013). Generalized Wang-Ball curve. Numer. Comput. Comput. Appl..

[19] Liu H.Y., Li L., Zhang D.M. (2011). Quadratic Ball curve with shape parameters. J. Shandong Univ..

[20] Huang C.L., Huang Y.D. (2012). Quartic Wang-Ball curve and surface with two parameters. J. Hefei Univ. Technol..

[21] Hu G., Zhu X.N., Wei G., Chang C.T. (2021). An improved marine predators algorithm for shape optimization of developable Ball surfaces. Eng. Appl. Artif. Intell.

[22] Hu G., Li M., Wang X., Wei G., Chang C.T. (2022). An enhanced manta ray foraging optimization algorithm for shape optimization of complex CCG-Ball curves. Knowl. Based Syst..

[23] Gurunathan B., Dhande S. (1987). Algorithms for development of certain classes of ruled surfaces. Comput. Graph.

[24] Jaklič G., Žagar E. (2011). Curvature variation minimizing cubic Hermite interpolants. Appl. Math. Comput..

[25] Lu L.Z. (2015). A note on curvature variation minimizing cubic Hermite interpolants. Appl. Math. Comput..

[26] Zheng J.Y., Hu G., Ji X.M., Qin X.Q. (2022). Quintic generalized Hermite interpolation curves: Construction and shape optimization using an improved GWO algorithm. Comput. Appl. Math.

[27] Hu G., Wu J.L., Li H.N., Hu X.Z. (2020). Shape optimization of generalized developable H-Bézier surfaces using adaptive cuckoo search algorithm. Adv. Eng. Softw..

[28] Ahmadianfar I., Heidari A., Gandomi A.H., Chu X., Chen H. (2021). RUN beyond the metaphor: An efficient optimization algorithm based on Runge Kutta method. Expert Syst. Appl..

[29] Hu G., Du B., Wang X.F., Wei G. (2022). An enhanced black widow optimization algorithm for feature selection. Knowl. Based Syst..

[30] Wolpert D.H., Macready W.G. (1997). No free lunch theorems for optimization. IEEE Trans. Evol. Comput..

[31] Nematollahi A.F., Rahiminejad A., Vahidi B. (2020). A novel meta-heuristic optimization method based on golden ratio in nature. Soft Comput..

[32] Holland J.H. (1992). Genetic algorithms. Sci. Am..

[33] Storn R., Price K. (1997). Differential evolution–a simple and efficient heuristic for global optimization over continuous Spaces. J. Glob. Optim..

[34] Koza J.R. (1992). Genetic Programming: On the Programming of Computers by Means of Natural Selection.

[35] Li W.Z., Wang L., Cai X., Hu J., Guo W. (2019). Species co-evolutionary algorithm: A novel evolutionary algorithm based on the ecology and environments for optimization. Neural. Comput. Appl..

[36] Kirkpatrick S., Gelatt C.D., Vecchi M.P. (1983). Optimization by simulated annealing. Science.

[37] Rashedi E., Nezamabadi-Pour H., Saryazdi S. (2009). GSA: A gravitational search algorithm. Inf. Sci..

[38] Mirjalili S. (2016). SCA: A Sine Cosine Algorithm for Solving Optimization Problems. Knowl. Based Syst..

[39] Hashim F.A., Hussain K., Houssein E.H., Mai S.M., Al-Atabany W. (2020). Archimedes optimization algorithm: A new metaheuristic algorithm for solving optimization problems. Appl. Intell..

[40] Talatahari S., Azizi M., Tolouei M., Talatahari B., Sareh P. (2021). Crystal structure algorithm (CryStAl): A metaheuristic optimization method. IEEE Access.

[41] Salawudeen A.T., Mu’Azu M.B., Sha’Aban Y.A., Adedokun A.E. (2021). A novel smell agent optimization (sao): An extensive cec study and engineering application. Knowl. Based Syst..

[42] Rao R.V., Savsani V.J., Vakharia D.P. (2012). Teaching-Learning-Based Optimization: An optimization method for continuous non-linear large scale problems. Inf. Sci..

[43] Satapathy S., Naik A. (2016). Social group optimization (SGO): A new population evolutionary optimization technique. Complex Intell. Syst..

[44] Bikash D., Mukherjee V., Debapriya D. (2020). Student psychology based optimization algorithm: A new population based optimization algorithm for solving optimization problems. Adv. Eng. Softw..

[45] Bodaghi M., Samieefar K. (2019). Meta-heuristic bus transportation algorithm. Iran J. Comput. Sci..

[46] Yuan Y.L., Ren J.J., Wang S., Wang Z.X., Mu X.K., Zhao W. (2022). Alpine skiing optimization: A new bio-inspired optimization algorithm. Adv. Eng. Softw..

[47] Kennedy J., Eberhart R. Particle swarm optimization. Proceedings of the ICNN’95-International Conference on NeuralNetworks.

[48] Dorigo M., Blum C. (2005). Ant colony optimization theory: A survey. Theor. Comput. Sci..

[49] Mirjalili S. (2015). Moth-flame optimization algorithm: A novel nature-inspired heuristic paradigm. Knowl. Based Syst..

[50] Ma L., Wang C., Xie N.G., Shi M., Wang L. (2021). Moth-flame optimization algorithm based on diversity and mutation strategy. Appl. Intell..

[51] Wang C., Ma L.L., Ma L., Lai J., Zhao J., Wang L., Cheong K.H. (2023). Identification of influential users with cost minimization via an improved moth flame optimization. J. Comput. Sci..

[52] Mirjalili S., Mirjalili S.M., Lewis A. (2014). Grey Wolf Optimizer. Adv. Eng. Softw..

[53] Mirjalili S., Lewis A. (2016). The whale optimization algorithm. Adv. Eng. Softw..

[54] Heidari A.A., Mirjalili S., Faris H., Aljarah I., Mafarja M., Chen H.L. (2019). Harris hawks optimization: Algorithm and applications. Future Gener. Comput. Syst..

[55] Hayyolalam V., Kazem A.A.P. (2020). Black Widow Optimization Algorithm: A novel meta-heuristic approach for solving engineering optimization problems. Eng. Appl. Artif. Intell..

[56] Huang Q.H., Wang C., Yılmaz Y., Wang L., Xie N.G. (2023). Recognition of EEG based on Improved Black Widow Algorithm optimized SVM. Biomed. Signal Process Control.

[57] Dhiman G., Kumar V. (2019). Seagull optimization algorithm: Theory and its applications for large-scale industrial engineering problems. Knowl. Based Syst..

[58] Mirjalili S., Gandomi A.H., Mirjalili S.Z., Saremi S., Faris H., Mirjalili S.M. (2017). Salp swarm algorithm: A bio-inspired optimizer for engineering design problems. Adv. Eng. Softw..

[59] Wang C., Xu R.Q., Ma L., Zhao J., Wang L., Xie N.G. (2023). An efficient salp swarm algorithm based on scale-free informed followers with self-adaption weight. Appl. Intell..

[60] Abdollahzadeh B., Gharehchopogh F.S., Mirjalili S. (2021). African vultures optimization algorithm: A new nature-Inspired metaheuristic algorithm for global optimization problems. Comput. Ind. Eng..

[61] Agushaka J.O., Ezugwu A.E., Abualigah L. (2022). Dwarf mongoose optimization algorithm. Comput. Methods Appl. Mech. Eng..

[62] Trojovský P., Dehghani M. (2022). Pelican Optimization Algorithm: A Novel Nature-Inspired Algorithm for Engineering Applications. Sensors.

[63] Nitish C., Muhammad M.A. (2022). Golden jackal optimization: A novel nature-inspired optimizer for engineering applications. Expert Syst. Appl..

[64] Zhao W., Wang L., Mirjalili S. (2022). Artificial hummingbird algorithm: A new bio-inspired optimizer with its engineering applications. Comput. Methods Appl. Mech. Eng..

[65] Ramadan A., Kamel S., Hassan M.H., Ahmed E.M., Hasanien H.M. (2022). Accurate photovoltaic models based on an adaptive opposition artificial hummingbird algorithm. Electronics.

[66] Mohamed H., Ragab E.S., Ahmed G., Ehab E., Abdullah S. (2022). Parameter identification and state of charge estimation of Li-Ion batteries used in electric vehicles using artificial hummingbird optimizer. J. Energy Storage.

[67] Sadoun A.M., Najjar I.R., Alsoruji G.S., Abd-Elwahed M.S., Elaziz M.A., Fathy A. (2022). Utilization of improved machine learning method based on artificial hummingbird algorithm to predict the tribological Behavior of Cu-Al_2_O_3_ nanocomposites synthesized by In Situ method. Mathematics.

[68] Abid M.S., Apon H.J., Morshed K.A., Ahmed A. (2022). Optimal planning of multiple renewable energy-integrated distribution system with uncertainties using artificial hummingbird algorithm. IEEE Access.

[69] Yildiz B.S., Pholdee N., Bureerat S., Yildiz A., Sait S. (2021). Enhanced grasshopper optimization algorithm using elite opposition-based learning for solving real-world engineering problems. Eng. Comput..

[70] Wang Y.J., Su T.T., Liu L. (2018). Multi-strategy cooperative evolutionary PSO based on Cauchy mutation strategy. J. Syst. Simul..

[71] Hu G., Chen L.X., Wang X.P., Guo W. (2022). Differential Evolution-Boosted Sine Cosine Golden Eagle Optimizer with Lévy Flight. J. Bionic. Eng..

[72] Seyyedabbasi A., Kiani F. (2022). Sand Cat swarm optimization: A nature-inspired algorithm to solve global optimization problems. Eng. Comput..

[73] Hu G., Zhong J.Y., Du B., Guo W. (2022). An enhanced hybrid arithmetic optimization algorithm for engineering applications. Comput. Methods Appl. Mech. Eng..

[74] Wilcoxon F., Bulletin S.B., Dec N. (1992). Individual Comparisons by Ranking Methods.

[75] Derrac J., García S., Molina D., Herrera F. (2011). A practical tutorial on the use of nonparametric statistical tests as a methodology for comparing evolutionary and swarm intelligence algorithms. Swarm Evol. Comput..

[76] Mohamed A., Reda M., Shaimaa A.A.A., Mohammed J., Mohamed A. (2023). Kepler optimization algorithm: A new metaheuristic algorithm inspired by Kepler’s laws of planetary motion. Knowl. Based Syst..

[77] Li S.M., Chen H.L., Wang M.J., Heidari A., Mirjalili S. (2022). Slime mould algorithm: A new method for stochastic optimization. Future Gener. Comput. Syst..

[78] Zheng J., Ji X., Ma Z., Hu G. (2023). Construction of Local-Shape-Controlled Quartic Generalized Said-Ball Model. Mathematics.

[79] Hu G., Wang J., Li M., Hussien A.G., Abbas M. (2023). EJS: Multi-strategy enhanced jellyfish search algorithm for engineering applications. Mathematics.

[80] Hu G., Zhong J., Wei G., Chang C.-T. (2023). DTCSMO: An efficient hybrid starling murmuration optimizer for engineering applications, Comput. Methods Appl. Mech. Eng..

[81] Hu G., Yang R., Qin X.Q., Wei G. (2023). MCSA: Multi-strategy boosted chameleon-inspired optimization algorithm for engineering applications. Comput. Methods Appl. Mech. Eng..

